# A Model for Evaluating the Procedural and Effectiveness Dimensions of Public Participation in Protected Areas: The SysMAPP

**DOI:** 10.1007/s00267-026-02603-0

**Published:** 2026-08-29

**Authors:** Giovana Cioffi, Davis Gruber Sansolo, Javier García Sanabria

**Affiliations:** 1 Research Group of Environmental Planning and Coastal Management, Bioscience Institute - São Paulo State University, Infante Dom Henrique Square, São Vicente, Brazil; 2 Research Group on Integrated Coastal Zone Management, University of Cádiz, Doctor Gomez Ulla Avenue, Cádiz, Spain

**Keywords:** Participation, Protected areas, Monitoring, Assessment, Tool

## Abstract

Despite broad recognition of the role of public participation in protected area (PA) success, persistent barriers highlight the need for tools to monitor and assess participatory processes and outcomes. This study proposes the System for Monitoring and Assessment of Public Participation (SysMAPP), designed to evaluate public participation in PAs by linking procedural conditions and management outcomes. The study is based on a systematic review of existing monitoring and assessment methods, the design of SysMAPP, and its application to PAs in Brazil and Spain. SysMAPP comprises five hierarchically organized, indicator-based criteria: Planning and Representativeness form the foundation of participation; Transparency and Influence connect information flow, accountability, and decision-making power; and Effectiveness captures management outcomes. Crucially, the model unveils the internal logic of participation by demonstrating how these criteria are causally and hierarchically related, showing that procedural and foundational quality directly condition substantive outcomes. SysMAPP also combines standardized core indicators with context-sensitive variables, enabling comparison across protected areas while preserving local governance specificities. Its application revealed contrasting participatory scenarios: strong stakeholder influence in Brazil, primarily through the incorporation of artisanal fisheries agendas, and weak participation in Spain, characterized by the limited representativeness of key groups such as shellfish gatherers and government-centered decision-making. Ultimately, SysMAPP advances beyond traditional scoring by operating as a diagnostic tool for adaptive governance; its results feed back into the management cycle through prioritized adaptation milestones, converting institutional diagnoses into targeted, context-sensitive interventions.

## Introduction

Protected areas (PAs) act as a relational mechanism connecting stakeholders, public authorities, and natural spaces (Haenssgen et al., 2021). By reshaping practices and expectations, PA regulation generates interpretations ranging from legitimacy to resistance; thus, negative social responses often reveal mismatches between policy and territory. Far from being isolated entities, PAs are embedded within geographic spaces where interactions among actors and their resource appropriation continuously produce multiple territories (Raffestin, 1993). This territorial configuration encompasses both a symbolic-cultural dimension of inscribed group identities and a material, politico-disciplinary dimension that orders space and people through a zonal logic; thus, spatial disputes are simultaneously conflicts over narratives, identities, and concrete domains (Haesbaert, 2007). Consequently, conflicts emerge when distinct modes of appropriation collide—typically when hegemonic actors like the State and large corporations treat the territory as an economic receptacle, opposing traditional groups whose territorial relations are grounded in subsistence and ancestral ways of life (Gottmann, 2012; Diegues, 2008). Participation thus is the pathway for articulation among these parties, shaping the meanings, uses, and desired futures of territories (Haenssgen et al., 2021). Depending on its configuration, participation can either reinforce power dynamics or shape patterns of inclusion and exclusion among different social groups (Jentoft et al., 2007; Haenssgen et al., 2021).

Addressing this relational complexity is therefore critical to PA governance, as the quality of these interactions directly dictates conservation success. Consequently, management effectiveness frameworks have been incorporating participation as a core analytical component. Ma et al. (2023)’s framework establishes a correlation between participation and better Indigenous Peoples and local communities (IPLCs) attitudes toward ecology and livelihoods and greater satisfaction with governance outcomes. Ospina et al. (2020) place participation within the “governance” category, linking it to outcomes and impacts, whereas Shields et al. (2016) treat participation and participatory outputs—consensus orientation and accountability—as conditions for decision legitimacy, reduced power asymmetries and conflict, lower costs, and enhanced social control. Kirkman et al.'s (2023) framework links the dimension of “stakeholder engagement” to “rights, access, and equity,” establishing that the latter cannot be achieved without the former. Other tools such as the Management Effectiveness Tracking Tool (METT) and the Rapid Assessment and Prioritisation of Protected Area Management (RAPPAM), follow the same logic (Leverington et al., 2010; Bialowolski et al., 2023).

While the importance of public participation for PA effectiveness is well established in the literature, improving arrangements beyond state-centered models remains necessary (Borrini-Feyerabend and Jaeger 2017). High‑quality and effective public participation in PA governance relies on core principles: power redistribution and real influence over decisions; inclusion and equity for vulnerable groups; interactive dialogue and deliberation between stakeholders and authorities; recognition of conflicts and power asymmetries; collective goal‑setting around a common good; sustained engagement; and diverse knowledge integration, from technical expertise to local perspectives (Arnstein, 1969; Rowe and Frewer 2000; Barragán Muñoz 2014). These principles converge with recent co-management scholarship. Synthesizing five decades of the field, Seijo Villamizar and García-Allut (2025) characterize co-management as a governance tool that redistributes power and shares responsibility among government, stakeholders and resource users, structuring a form of shared intelligence over public affairs. In their reading, participation gains substance when this power-sharing is coupled with social learning and the co-production of knowledge—bridging scientific and local expertise— thereby strengthening the institutional resilience and adaptive capacity of socioecological systems.

However, the literature reports persistent barriers to evolved participation. These include limited financial and human resources and restricted access to information (Dewan et al., 2014; Bockstael et al., 2016; Quesada‑Silva et al., 2019; Flannery et al., 2018; Seixas et al., 2019). Another recurrent challenge is the underrepresentation of stakeholders, particularly IPLCs, women, and other vulnerable groups (Dewan et al., 2014; Hovik et al., 2010; Nadeem and Fischer, 2011; Bockstael et al., 2016; Cioffi et al., 2024). Yet a more decisive barrier is institutional rather than technical or financial: the resistance of public authorities to share decision-making power and enable social control, which translates into inaction in promoting participation and implementing collectively agreed decisions (Barragán Muñoz, 2014; Ruiz-Villaverde and García-Rubio, 2017). As a result, formally participatory arenas can persist for decades while delivering little of the substantive influence they promise: committees are constituted, meetings are convened, minutes are recorded, and consultations are conducted, yet the decisions that matter continue to be made elsewhere (Parkins and Mitchell, 2005; Bawole, 2013; Hovik et al., 2010). As a result, participatory processes frequently translate into lacking or limited outcomes for protected areas (Barragán Muñoz, 2014; Cioffi et al., 2024). The central gap is, then, between the institutional form of participation and its capacity to shape PA outcomes—which is precisely why assessing real stakeholder influence, rather than formal participation alone, becomes essential.

This reality calls for specific tools to monitor and assess participation to support its improvement. Existing monitoring and assessment (MA) frameworks within environmental management are generally bifurcated into process and outcome evaluations. Process evaluation focuses mainly on methodological and structural aspects to respond to how participation is implemented (Nadeem and Fischer, 2011; Carr et al., 2012). Outcome evaluations, in turn, examine intermediate results (e.g., agreements and cooperation initiatives) and impacts on environmental conditions, social dynamics, and human activities. Measuring impacts on socioecological conditions remains a major challenge for MA tools, as these impacts involve long-term changes and are difficult to directly attribute to participatory processes. By contrast, intermediate results, while not representing final conservation goals, are critical to achieving them and are more measurable within analytical models (Carr et al., 2012). However, the existing frameworks tend to prioritize the evaluation of procedural aspects over process–effect relationships (Olsen et al., 2009; Nadeem and Fischer, 2011; Carr et al., 2012).

To bridge the gap between procedural quality and outcome achievement, integrated tools are needed that offer a holistic view of the participatory process. It is this gap that this study addresses. To that end, this study proposes the System for Monitoring and Assessment of Public Participation (SysMAPP), designed to monitor and assess both the processes and outcomes of public participation in protected areas, supporting PA managers in reviewing and adapting participatory processes and strengthening social control over them. The work presents a systematic literature review (SLR) of existing MA tools, describes the development of SysMAPP, and presents its application in PAs in Brazil and Spain. SysMAPP is expected to contribute on four fronts: enhancing institutional efficiency through participation goal setting and results monitoring; strengthening PA effectiveness by tracking cause–effect relationships between procedures and outcomes; promoting citizenship and rights through institutional–social alignment; and providing evidence to counter recurrent administrative objections to participation, such as increased costs or decision-making delays

Within this framework, the study addresses two research questions: (RQ1) How can an MA model be designed to maintain analytical rigor while adapting to the diverse socioecological contexts of public participation? (RQ2) How can the systematic MA of public participation support the legitimacy and adaptive management of PAs? Because participation is context-dependent yet shares recurrent features across PAs (Rowe and Frewer 2000), the study examines two hypotheses: H1—an MA system can reconcile standardized core indicators with site-specific variables, supporting both contextual adaptability and cross-case comparison; H2—jointly assessing participatory procedures and their outcomes can reveal the conditions under which participation strengthens PA legitimacy and adaptive management.

## Methodology

### The Design Process of SysMAPP

**Fig. 1:**
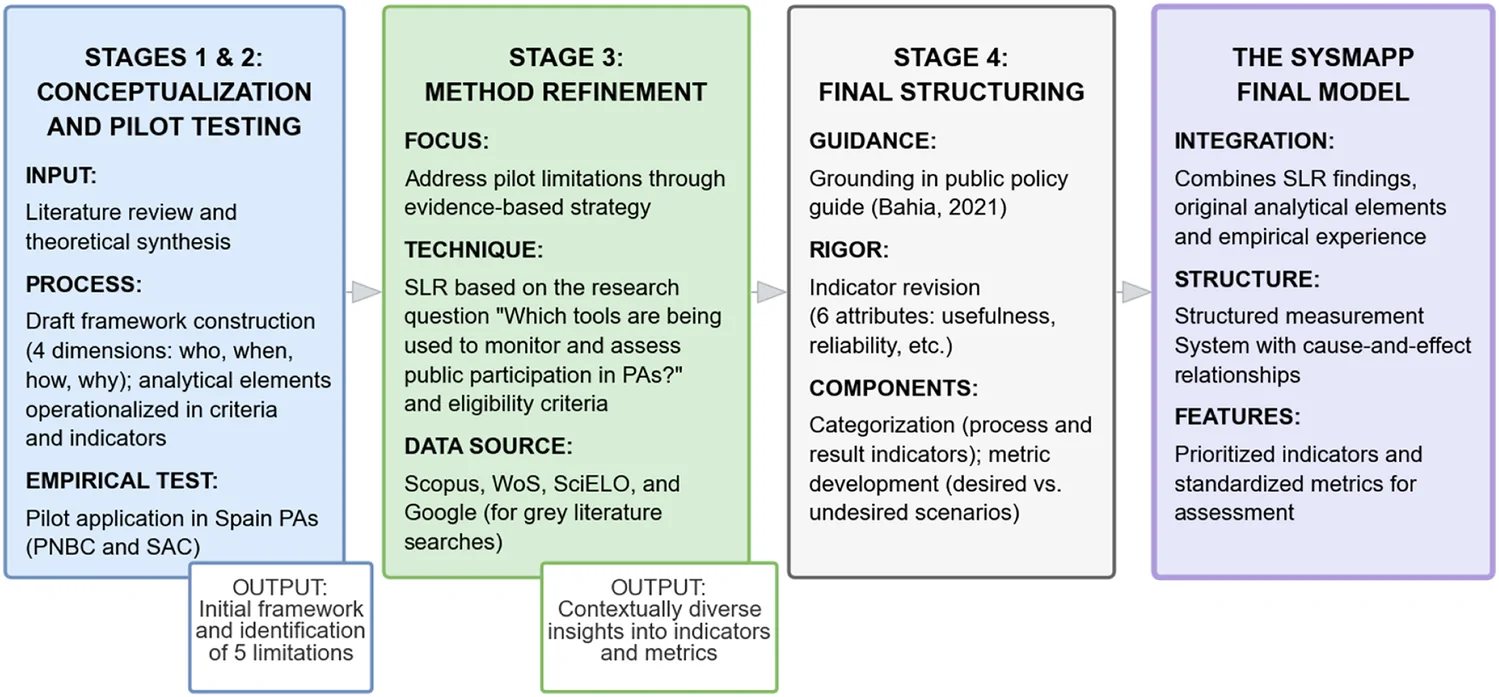
Overview of the four stages that led to the development of the final SysMAPP, as detailed in the methodology section

The development of the SysMAPP followed a four-stage evolutionary design (Fig. 1). The model is composed of five criteria, each measured through a set of indicators. The process integrates theoretical synthesis, preliminary empirical testing, and a systematic literature review (SLR) to ensure a comprehensive construction of the system by gathering analytical elements on participation applied in different contexts worldwide.

#### Stages 1 and 2—Conceptual Framework, Preliminary Indicator Selection and Testing

The first stage established the conceptual basis of the system through a literature review. The searches were conducted in Scopus and Web of Science using keywords such as “stakeholder/public participation,” “evaluation/assessment,” and “protected areas/environmental planning”. Three primary criteria were applied to filter the results: (a) review articles focused on evaluation methods; (b) studies proposing new models; and (c) empirical case studies.

The analytical elements identified in the literature were categorized into four dimensions—who (who participates?), when (at which stage?), how (through which strategy?), and why (for what purpose?) (Morf et al., 2019). Subsequently, they were operationalized in the form of criteria and indicators (Ind.), following Brand's (1997) definition, in which criteria are foundational components, while indicators are their measurable properties. Once selected, the criteria and indicators were submitted for consultation and validation by eight experts from Brazil, Spain, and Portugal. The experts were nominated by the supervisors of this work based on their recognized expertise in public participation and protected-area governance; the final number reflects those identified as relevant who were both available and willing to participate, rather than a predetermined sample size.

For each criterion, an evaluative question was formulated to guide the selection and interpretation of its indicators. These questions were not defined a priori: they were derived from the theoretical review conducted in this stage, each synthesizing what the literature expects the corresponding criterion to achieve. The Planning question, for instance, draws on the principle that participation must be accessible and clearly defined in its scope, rules, and resources (Barragán Muñoz, 2014; Rowe and Frewer, 2000; Carr et al., 2012). Each question thus translates the theoretical purpose of its criterion into an operational evaluative focus; the full theoretical grounding of every criterion is detailed in Section 3.1.1.

A pilot application (stage 2) was conducted in two PAs in Spain: the Bahía de Cádiz Natural Park (PNBC) and the Cadiz Bay Seabed Special Area of Conservation (SAC). The results of the pilot application were published in the journal Ocean and Coastal Management. The pilot application revealed five methodological limitations, presented in Table 1 together with the criteria to which they relate.

**Table 1:** Limitations identified in the pilot application, corresponding corrective actions, and the associated criteria

Detected limitation (pilot)	Change introduced in SysMAPP	Affected criterion
State-centered focus: assessment centered on public authorities, neglecting non-state stakeholders’ own commitment	Added indicators capturing which segments participate actively and members’ commitment/attendance	Representativeness and Influence
No prioritization of indicators: equal weighting hindered resource allocation for adaptation	Introduced a hierarchy of mandatory/priority vs. non-mandatory indicators, feeding the scoring rule and adaptation milestones	Cross-cutting (scoring logic)
Thematic gaps: no indicator for the relevance of debated content	Added assessment of whether debated topics are pertinent to PA objectives	Effectiveness
Excessive subjectivity: vague indicators compromised replicability	Redesigned indicators to be concretely traceable; refined nomenclature	Cross-cutting (all criteria)
Lack of metrics: no standardized interpretation	Developed best/worst-scenario metrics on a 0–3 ordinal scale	Cross-cutting (scoring system)

#### Stage 3—Method Refinement Through Systematic Literature Review

The SLR followed the protocol of CEE (2013) and Pegler et al. (2024) and was guided by a closed-frame question: “Which tools are being used to monitor and assess public participation in protected areas?”. Whereas the conceptual review in Stage 1 had been restricted to Scopus and Web of Science, the SLR added SciELO to improve coverage of Latin American methods, and Google was used to retrieve gray literature (guides, reports, dissertations, theses, and books). Searches were run in English, Portuguese, and Spanish. The complete search strings are reported in Table 2; in each language, they combined three structural blocks—assessment terms AND participation terms AND protected area terms. For gray literature, the first three results pages of each Google search were screened.

**Table 2:** SLR search strings

Language	Assessment terms	Participation terms	Protected-area terms
English	“assessment” OR “monitoring”	“public participation” OR “stakeholder participation”	“protected area”
Portuguese	“análise” OR “monitoramento”	“participação pública” OR “participação social” OR “participação comunitária”	“áreas protegidas” OR “unidades de conservação”
Spanish	“evaluación” OR “monitoreo”	“participación pública” OR “participación ciudadana”	“áreas protegidas” OR “espacios naturales protegidos”

To reduce selection bias, a research protocol with explicit eligibility criteria was established and rigorously followed. The criteria were: (i) language (English, Portuguese, or Spanish); (ii) full-text availability; (iii) research area (environmental science); (iv) scope (empirical studies or methodological proposals addressing participation, including PA-effectiveness methods that contained participation components); and (v) study area (terrestrial or marine PAs). The content of the included documents was then systematized in a structured matrix with predefined extraction categories—bibliographic and contextual data (authors, year, source, country), type of document, type of assessment tool, participation focus (process and/or outcome), analytical elements of participation identified, the SysMAPP criterion each element informed, and the metric or scoring approach used.

The database searches initially yielded 2977 records (720 from Scopus, 2234 from Web of Science, and 23 from SciELO), which were reduced to 1554 after removing duplicates in Mendeley. Applying the eligibility criteria narrowed this down to 52 peer-reviewed studies, while a gray-literature search added 34 documents—primarily assessment and monitoring guides (18) and reports (8). This resulted in a total of 86 reviewed documents, mostly published after 2010 (96%) and geographically concentrated in Latin America, followed by Europe and Asia. The high number of exclusions was due to a lack of alignment with the research scope, primarily comprising health-related studies (31%) and environmental research outside the evaluation of public participation (e.g., biodiversity monitoring, ecosystem-services valuation, water-quality and pollution studies, habitat mapping, climate-change impacts, and tourism).

#### Stage 4—Final Structuring of SysMAPP

The operationalization of the SLR findings into criteria and indicators (Stages 3–4) was carried out by the authors; expert involvement was confined to Stage 1. The final structure of SysMAPP indicators adopts, as its methodological basis, the published reference guide for the construction and analysis of public-management indicators of Bahia (2021), which frames a set of indicators as a measurement system sustained by cause-and-effect relationships. Following Bahia’s (2021) rigor, each indicator was selected based on six attributes: usefulness for decision-making; representation of the measured phenomenon; methodological reliability and reliable data sources; cost-effectiveness; simplicity of understanding and application; and stability with minimal external interference.

To ensure high empirical applicability, only operationally feasible metrics were retained from the SLR pool, while abstract, overly theoretical, or excessively data-intensive variables were excluded. This filtering kept the system compatible with the information routinely available in PAs (e.g., management plans and meeting records), preventing the methodology from becoming too complex or resource-heavy for practical governance.

Indicators were defined directly from the SLR evidence base. Each participatory element reported across the reviewed methods was extracted as a candidate variable, screened against the six attributes above, and clustered with functionally related variables to form a single, measurable indicator. For example, several variables describing a well-defined legal framework—participation objectives, rules, meeting format, and the like—were consolidated into a single indicator assessing the institutional framework of participation. This clustering by analytical affinity was applied across all five criteria to yield a streamlined, traceable, multi-dimensional index. The full correspondence between the SLR sources and each resulting indicator is provided in supplementary data. Metrics were developed to represent best- and worst-case performance scenarios informed by the theoretical framework, thereby defining realistic desired and undesired conditions (Bahia 2021).

Within this final set, a subset of indicators was designated as priority: those judged structurally more important than the others to achieve participation quality, while the remaining (non-priority) indicators stay analytically relevant. Their identification rested on the authors’ own analysis of what constitutes high-quality participation, developed through continuous discussion within the authors’ research groups and informed by the recurrence of elements across the SLR (those appearing in most of the reviewed methods) and by the main insights from the cross-sector interviews.

### Contextualization of the PAs for the SysMAPP Application

**Fig. 2:**
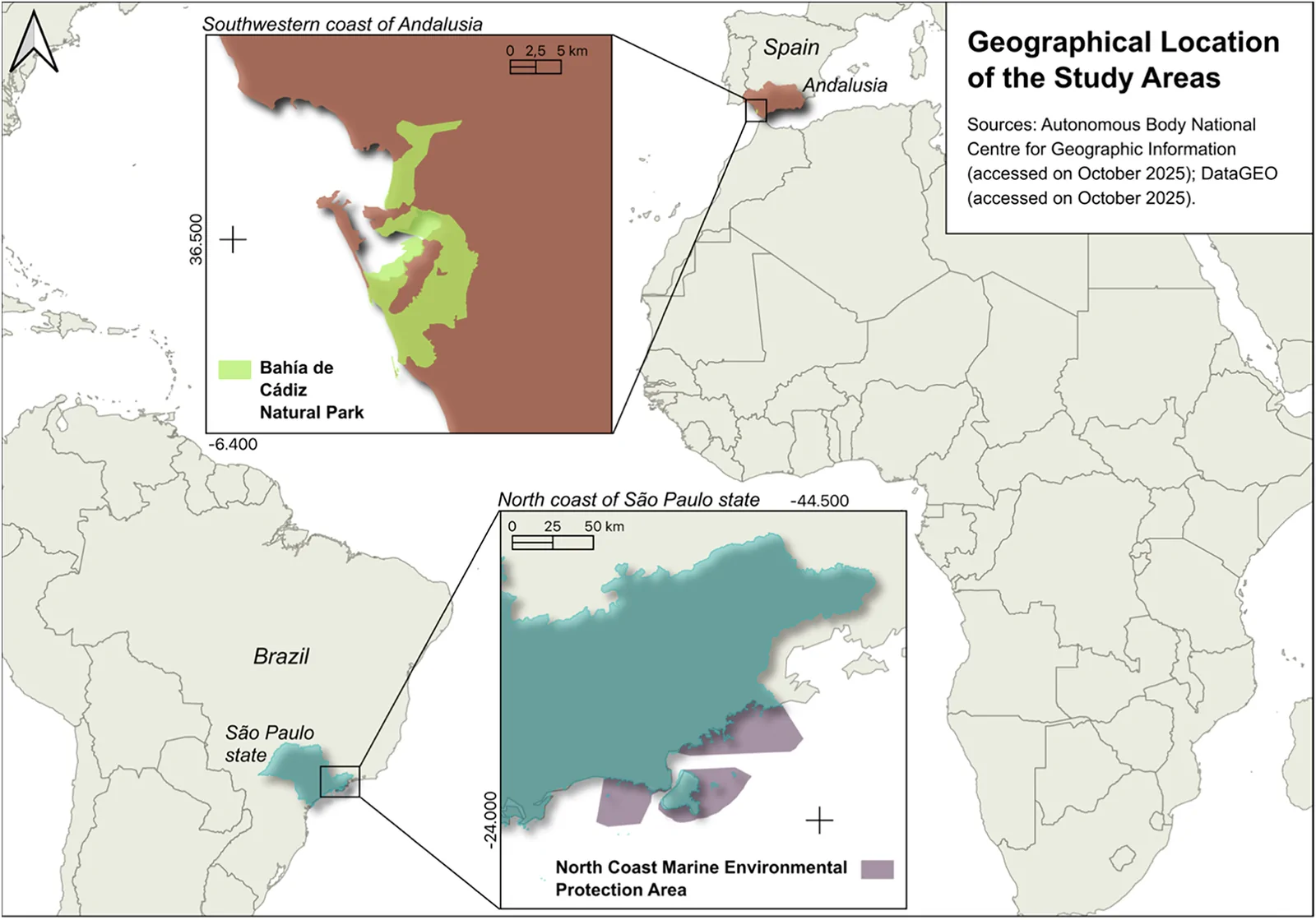
Geographic location of the protected areas selected as case studies in Brazil and Spain

The final SysMAPP version was applied to two coastal PAs classified under IUCN Category V—protected landscapes/seascapes, a sustainable-use category in which conservation is pursued together with regulated human use rather than strict protection (Fig. 2): the North Coast Marine Environmental Protection Area (APAMLN), in Brazil, and the Bahía de Cádiz Natural Park (PNBC), in Spain. Despite their different designations, both are multiple-use, human-modified seascapes where regulated economic activity coexists with conservation, which makes public participation central to their governance.

In the Brazilian system, the Environmental Protection Area is within the sustainable-use category defined by the National System of Conservation Units (SNUC, Law 9.985/2000). Created in 2008 (Decree 53.525/2008) to regulate recreational tourism, research, and fishing, the APAMLN spans roughly 3150 km² across four municipalities (Ubatuba, Caraguatatuba, Ilhabela, and São Sebastião) and is organized into three sectors. As a low-restriction category, it does not extinguish prior land- and sea-uses but overlays them with management rules; it extends seaward to the 50 m isobath and overlaps the state Ecological-Economic Zoning (EEZ) up to its 23.6 m isobath limit. Its binding spatial-planning instrument is the management plan (finalized in 2022), which sets the zoning and use norms required by SNUC and is mediated through the consultative council.

In the Spanish-Andalusian system, the Natural Park is likewise a sustainable-use figure intended to protect areas of high ecological value while sustaining traditional socioeconomic activity. Established in 1989 (Law 2/1989) and managed by the Regional Ministry of the Environment of the Andalusian Government, the PNBC covers 105 km² of predominantly intertidal ecosystems (salt marshes and salt pans) shaped by two millennia of salt production and extends across five municipalities. It coexists with a denser set of planning instruments: a Natural Resource Management Plan (PORN) and a Master Use and Management Plan (PRUG) (both in their second version, approved in 2024) which define zoning and use rules, complemented by a Sustainable Development Plan (revised in 2022). The area is further integrated into the EU Natura 2000 network, as a Special Protection Area for birds and bordered by a marine Special Area of Conservation.

The two study areas differ in territorial scale and in their sociocultural and political contexts. The Brazilian case is shaped by a long trajectory of environmental democratization and by the central role of Indigenous Peoples and traditional communities, including the recognition of customary rights in PA management (Jacobi, 1996; São Paulo Government, 2022), whereas the Spanish case follows a different path, marked by lower levels of participatory engagement in environmental affairs (Barragán Muñoz and Ruiz, 2015).

Despite these contrasts, both share the features that justify their joint analysis: they are coastal zones of high ecological and cultural value where multiple modes of appropriating the territory overlap and frequently collide. In both, unregulated tourism and associated real-estate speculation coexist with large-scale activities—ports, shipping and, in the Brazilian case, oil and gas exploitation—whose cumulative socioecological impacts heighten decision-making complexity. In the APAMLN, port and oil-and-gas activities conflict directly with traditional livelihoods (mainly small-scale fishing) through marine pollution and the consequent decline in fish stocks, competition for space with large vessels, accident risks and damage to fishing gear, and the restriction of fishing grounds by anchorage zones, infrastructure and port expansion (São Paulo Government, 2022). In the PNBC, marked land-use change between 1956 and 2016—with urban areas, industry, tourism and port infrastructure all expanding several-fold (tourism installations by roughly 78 times and industry by about 14 times)—has driven habitat loss (mainly of the saltmarsh ecosystem) and pollution (Barragán Muñoz and de Andrés, 2020; Andalusian Government, 2024). Crucially, both areas host vulnerable groups—artisanal fishers in Brazil, shellfish gatherers and salt workers in Spain—whose livelihoods are squeezed at once by conservation restrictions and by these hegemonic uses, a double bind that reproduces and deepens their vulnerability.

The SysMAPP is structured around five core criteria: Planning, Representativeness, Transparency, Influence, and Effectiveness. Each criterion addresses specific participation gaps identified in previous studies on PAs governance. The operationalization of criteria based on the SLR findings is detailed in the following sections.

## Results and Discussion: Building and Testing the SysMAPP

### Translating SLR Findings into the SysMAPP Analytical Framework

Participation and governance assessment are conceptually interrelated: participation is widely treated as a constitutive dimension of good governance (Graham et al., 2003; Shields et al., 2016), while governance is the broader frame that conditions how—and how far—participation is exercised. This relationship is reflected in the reviewed corpus, which spans two complementary research traditions. The first (≈30% of the corpus) evaluates the overall effectiveness and governance of PAs, where participation appears as one component of a wider assessment, typically within a “management and governance” axis—terms often used interchangeably or nested in one another (e.g., management as a subfactor of governance in Huang et al., 2024). The second (≈70%) focuses specifically on participatory processes and their effects, examining them in detail. As outlined in the Introduction, effectiveness and governance frameworks position participation along a spectrum—from a procedural instrument of management (e.g., METT, RAPPAM) to a central, outcome-linked driver (e.g., Ospina et al., 2020; Kirkman et al., 2023)—whereas the participation-specific literature supplies the granular procedural and outcome indicators that those frameworks tend to leave implicit.

Across the corpus, the two traditions also differ in their degree of consolidation. PA-effectiveness frameworks are the most consolidated and the most frequently embedded in public policy: several are issued or adopted by governments and conservation agencies (e.g., PROFONANPE 2007 in Peru, Ecuador Environmental Ministry 2015, SINAC 2016 in Costa Rica), and standardized instruments such as METT (Leverington et al., 2010) and RAPPAM (WWF, n.d.) have been applied at a global scale (Leverington et al., 2010), across the terrestrial and marine PAs covered by this review. Their main contribution lies precisely in this breadth, comparability, and institutional uptake. Within them, however, participation is typically reduced to a single procedural item on a broad checklist—treated as a means to management success rather than as an object of assessment in its own right (a notable exception being Ecuador Environmental Ministry, 2015, where participation figures as a management programme to be delivered)—so they capture neither the quality of the participatory process nor its influence on decisions and outcomes.

Tools that focus specifically on participation are dispersed across at least five distinct and often overlapping objectives: assessing the legal design and implementation of participation (≈35%), frequently centered on managing councils (e.g., Ávila et al., 2005; Oliveira et al., 2023) or sector-specific arenas such as fisher engagement (e.g., García and Méndez 2019); developing or testing assessment methods (≈27%; e.g., Carrillo et al., 2023; Sáenz-Chávez et al., 2020); evaluating a single participatory planning or management process in depth (≈23%; e.g., Kovács et al., 2017; De Santo 2016); mapping stakeholders, their networks and perceptions (≈10%; e.g., Rastogi et al., 2010; Calvet-Mir et al., 2015); and gauging the effect of participation on social acceptance and PA performance (≈6%; e.g., Rees Catalán 2015; Dalton et al., 2012). Even within the method-development strand, proposals tend to be confined to a single dimension—for instance, minutes analysis (e.g., Dilascio et al., 2023) or official websites (e.g., Rossi et al., 2024) used as proxies for deliberation or transparency—and seldom combine procedural and outcome assessment. This contrast—consolidated-but-shallow effectiveness tools versus detailed-but-dispersed participation tools—is precisely the gap SysMAPP addresses.

The mapping of analytical elements followed a deductive coding procedure. Only studies presenting an explicit list of indicators or analytical variables were considered at this stage; each item was extracted into a matrix recording the authors, the dimension or criterion used in the original study, the evaluative question, the indicator or variable, and the SysMAPP criterion to which it was assigned. Items were classified against the five pre-established criteria according to their content and explanatory alignment with each criterion’s definition; those that were overly subjective or generic, or that did not fit a single criterion (e.g., “the intrinsic value of nature is respected”), were coded as not applicable. The procedure mapped 407 indicators and variables, of which 358 were assigned to the five criteria, and 49 were coded as not applicable.

**Fig. 3:**
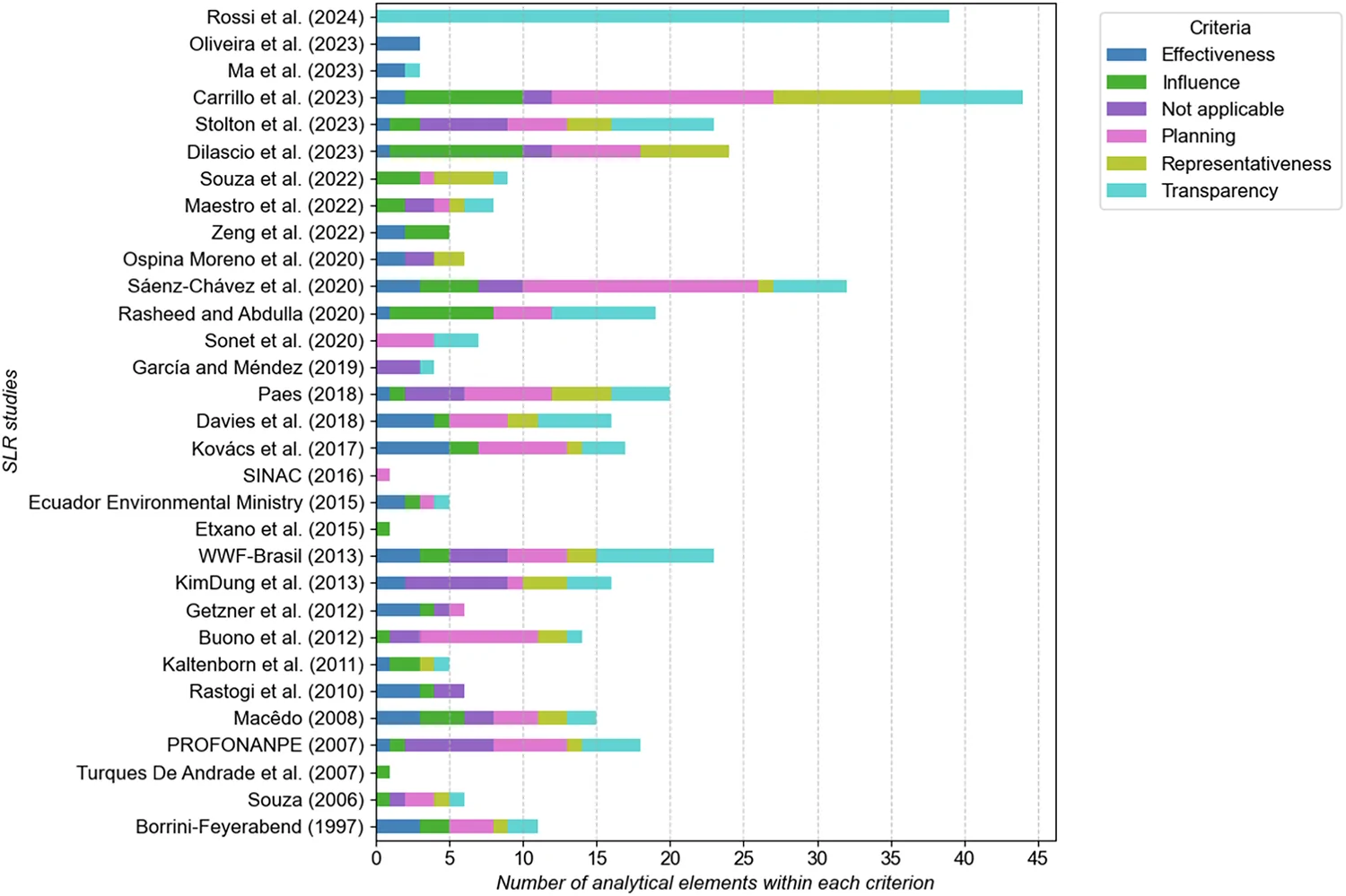
Analysis of the number of analytical elements corresponding to each SysMAPP criterion by SLR study

Consistent with Carr et al. (2012) and Olsen et al. (2009), most tools identified in the SLR emphasize procedural aspects (“how” participation occurs), while outcome‑oriented indicators are less common. Among analytical elements mapped to SysMAPP criteria (Fig. 3), transparency (108) and planning (96) predominate, followed by influence (59), effectiveness (48), and representativeness (47). Overly subjective or generic elements (e.g., “respect for the intrinsic value of nature”) were excluded as not applicable. A systematized version of the analytical elements, obtained by aggregating similar items, is provided in the supplementary data.

#### Operationalizing Participation Quality Through Five Assessment Criteria

##### Planning: institutionalizing the participatory arena

The planning criterion evaluates the degree of organization, legal framing, and procedural accessibility to answer the evaluative question “Is the participation process structured and regulated to ensure an accessible and legitimate environment for debate?” Theoretically, a structured framework reduces ambiguity and mitigates conflicts by addressing multi-stakeholders’ expectations and resource accessibility (Rowe and Frewer, 2000; Barragán Muñoz, 2014). Beyond logistics, this criterion focuses on institutional enfranchisement by establishing the legal foundations necessary for the systematic monitoring and accountability of the process itself (Rowe and Frewer, 2000; Nadeem and Fischer, 2011; Carr et al., 2012; Quesada-Silva et al., 2019). The institutionalization of participation architecture prevents the participatory “action space” from being overlooked or co-opted by dominant interests, by ensuring a continuous, informed and inclusive debate (Buanes et al., 2005; Carr et al., 2012).

The results of the SLR showed that indicators within the Planning are bifurcated into two dimensions. The first covers the institutional architecture: legal frameworks to formalize objectives, responsibilities, and rules of the participatory process (Borrini-Feyerabend, 1997; Macêdo, 2008). The second focuses on the infrastructure of the “action space,” assessing meeting frequency, location, and form, the existence of thematic sub-committees (Paes, 2018; Sáenz-Chávez et al., 2020), and the allocation of resources—including budget, qualified facilitators, and diverse participatory methods (Buono et al., 2012; Sonet et al., 2021). A specific subset targets the inclusion of vulnerable groups through logistical adjustments like transport and flexible scheduling (Buono et al., 2012; Paes, 2018; Souza, 2006). Finally, planning encompasses self-monitoring mechanisms, including periodic evaluations and procedures to ensure compliance with agreements (Carrillo et al., 2023).

##### Representativeness: defining whose interests enter the arena

Representativeness is the cornerstone of public acceptance and process legitimacy, as it assesses whether the participatory arena mirrors the territory’s social diversity (Rowe and Frewer, 2000). It seeks to answer: Does the participatory arena mirror the social diversity and ensure the effective exercise of representation for all segments? Theoretical frameworks suggest that key stakeholders can be identified by their land rights, historical ties, resource dependency, possession of management-essential expertise, and potential impact on conservation goals (Borrini-Feyerabend, 1997; Marinelli, 2014; Hovik et al., 2010; Morf et al., 2019). Representativeness accounts for the “influence-importance” paradox (UNDP, 2009); those groups most impacted by management decisions often possess the least capacity to influence them. This criterion evaluates the institutional commitment to mapping and recognizing vulnerable groups, ensuring their inclusion transcends nominal representation to become a prioritized and practiced element of daily PA governance.

Assessing Representativeness depends on four key analytical aspects identified in the reviewed studies. First, concerns the breadth and diversity of the participatory arena. It includes specific and operational indicators such as the number of representatives, diversity of institutions or sectors (Souza et al., 2022; Dilascio et al., 2023), compositional parity (WWF-Brasil, 2013), and the number of represented institutions (Dilascio et al., 2023). The second relates to accountability in the exercise of participation. It is mainly composed of more generic variables, for example, “council members act with commitment” (Paes, 2018; Borrini-Feyerabend, 1997) or proxy indicators such as attendance frequency at meetings (Dilascio et al., 2023). Third is perceived legitimacy, capturing how stakeholders recognize the arena as a democratic representative of their rights (Paes 2018). The fourth involves a more complex analysis of territorial configuration, assessing whether council representation is proportional to stakeholders’ degree of involvement or impact within the protected area (Carrillo et al., 2023).

##### Transparency: enabling information flow and accountability

Transparency serves as permanent light over the participatory chain, aiming to answer: “Is transparency guaranteed through documentation and the democratization of information?” (Rowe and Frewer, 2000; Nadeem and Fischer, 2011; Bockstael et al., 2016). Theoretically, its primary function is to mitigate mistrust and enable social control. In this sense, Transparency operates in synergy with Planning: while Planning defines the normative “should-be” of the process, Transparency provides the empirical evidence of “how it is” by ensuring the availability of raw data. In this framework, information democratization transcends the disclosure of administrative acts and scientific data; it demands linguistic accessibility through clear, non-technical, and multilingual communication (UN, 2023). By ensuring that information is not only available but also comprehensible, transparency prevents the marginalization of non-specialist actors and allows for the effective monitoring of how proposals are considered, justified, and integrated into PA management.

Transparency-related elements can also be grouped into four complementary aspects, according to SLR findings. The first concerns the visibility of the participatory process, assessed through access to prior information on meetings and activities, as well as the documentation, dissemination, and validation of records and minutes (KimDung et al., 2013; Carrillo et al., 2023). The second refers to the availability of supporting data in public repositories (e.g., diagnostics, zoning plans, environmental impact studies, etc.) (Maestro et al., 2022; Rossi et al., 2024; Davies et al., 2018; Macêdo, 2008; PROFONANPE, 2007). Third encompasses dialogue and feedback mechanisms, such as communication channels and protocols that clarify how decisions are made and implemented (Sáenz-Chávez et al., 2020; Paes, 2018; Souza et al., 2022; Carrillo et al., 2023; Rossi et al., 2024). Finally, accountability includes formal complaint channels, budget transparency, and the systematic disclosure of information on conflicts and litigation (Borrini-Feyerabend, 1997; Rossi et al., 2024; Rasheed and Abdulla, 2020).

##### Influence: assessing the redistribution of decision-making power

Influence measures the degree to which stakeholders exercise effective decision-making power, answering the question: “To what extent do stakeholders exert influence over the management of the PA?” Influence is the primary driver of long-term engagement; participants only recognize value in the process when they perceive that their contributions lead to concrete assimilation (Rowe and Frewer, 2000; Nadeem and Fischer, 2011; Cioffi et al., 2024). Consequently, this criterion assesses the extension of power—identifying whether stakeholder reach is restricted to operational consultations or if it encompasses strategic policy and budgetary control (Barragán Muñoz, 2014). In the context of PAs, meaningful influence must extend over fundamental dimensions such as objectives, zoning, manager election, and financial allocation (Loureiro and Cunha, 2008). Crucially, legitimacy depends heavily on building an epistemic consensus through interactive networks and the co-production of knowledge, rather than merely offering spaces for consultation. This approach transforms governance into a collaborative method for sharing power, transferring responsibility across levels, and structuring shared intelligence to solve complex contextual problems (Seijo Villamizar and García-Allut 2025). Without sharing power over matters critical to the PA, participation risks becoming a symbolic exercise used to legitimize predetermined state decisions (Arnstein, 1969; Barragán Muñoz, 2014; Cioffi et al., 2024).

Influence indicators often rely on proxies or qualitative variables in the SLR studies. The assessment of interaction quality in participatory arenas tends to rely on abstract measures: “respect and attention to different viewpoints” (Souza et al., 2022), “opportunities for dialogue” or “absence of devaluing verbal or non-verbal behaviors” (Rasheed and Abdulla, 2020). The treatment of proposals by the public authority ranges from broad indicators, such as “the forwarding of deliberations according to represented sectors,” to more objective metrics, including the “proportion between proposals presented and incorporated” (Turques de Andrade et al., 2007; Kovács et al., 2017) and the “number of deliberations per meeting” (Dilascio et al., 2023). Decision-making authority and process autonomy are assessed through variables related to the process nature (deliberative versus consultative) (Souza, 2006), occurrence of decisions independent of PA management preferences, stakeholders’ control over council composition and structure (Carrillo et al., 2023; Getzner et al., 2012), and participation in zoning, management plans etc. (Zeng et al., 2022; Maestro et al., 2022; Macêdo 2008; Sáenz-Chávez et al., 2020), participative agenda-setting (Dilascio et al., 2023) and stakeholders’ perceptions of power dynamics and effective influence (Kaltenborn et al., 2011).

##### Effectiveness: linking participation to intermediate governance outcomes

Effectiveness evaluates the generation of intermediate results (Carr et al., 2012) to answer “Has public participation generated results, both for PA objectives and for broader social trust?”. Rather than measuring socioecological impacts, this criterion focuses on the procedural outputs that increase the likelihood of PAs sustainability. The achievement of long-term sustainability depends on a sequence of participatory effects that encompass increasing social awareness of PAs (Dilascio et al., 2023), constructing mutual trust between stakeholders and management (Barragán Muñoz, 2014), facilitating joint actions or overcoming impasses by identifying common ground (Van den Hove, 2006), promoting policies and practices tailored to the local context (Connick and Innes, 2003; Petts, 2006). By mapping these outcomes, it becomes possible to link process quality to performance and justify the cost–benefit ratio of participatory investments (Rowe and Frewer, 2000).

Regarding Effectiveness, a central emphasis is placed on social support, with perception-based indicators (e.g., “does the community support management?”—Ma et al., 2023; council members’ assessment of the council’s usefulness—Carrillo et al., 2023). There are also variables to measure participation effects on management, such as conflict handling, agreements implementation (Sáenz-Chávez et al., 2020), number of agreements per meeting (Dilascio et al., 2023), and agreements expansion over time in geography and complexity terms (Borrini-Feyerabend, 1997). Indicators related to social learning and knowledge integration are also prominent, including multidirectional learning (Davies et al., 2018; Paes, 2018), traditional and scientific knowledge combining (Davies et al., 2018; Kovács et al., 2017), and increased awareness of PAs (Dilascio et al., 2023; Rastogi et al., 2010). Moreover, some variables were identified to capture collective responses and cooperation, such as joint monitoring actions (Zeng et al., 2022) and the design of new policies, incentives, and compensation mechanisms (Kovács et al., 2017; Macêdo, 2008).

#### From Indicators to Scoring Scenarios: the SysMAPP Assessment Logic

Regarding metrics, the SLR revealed four predominant approaches to interpreting indicators: (i) binary metrics (Carrillo et al., 2023; Rossi et al., 2024); (ii) Likert scales (Oliveira et al., 2023; Paes 2018); (iii) multi-interval ordinal scales (PROFONANPE 2007; KimDung et al., 2013; Ecuador Environmental Ministry 2015; Kovács et al., 2017; Macêdo, 2008; Zeng et al., 2022; Maestro et al., 2022; Carrillo et al., 2023); and (iv) descriptive qualitative categories (Sáenz-Chávez et al., 2020; Stolton et al., 2021). Among these approaches, SysMAPP employs an ordinal scale enabling precise and flexible assessment of participatory processes. Scores range from 0 to 3 (red to blue), indicating from absence to full achievement of the best scenarios. To standardize interpretation, the system categorizes performance into four levels: Critical, Deficient, Improved, and Advanced (Table 3).

**Table 3:** Classification matrix for SysMAPP scenarios, general thresholds, and borderline transition zones

Scenario	General Threshold	Boundary Factors (Transition Zones)
**Advanced**	Most indicators score 3, including all priority indicators.	Borderline Advanced: Most indicators score 3, but one or more priority indicators score 2, placing the arena at risk of dropping to Improved.
**Improved**	Most indicators score 2 or above, including all priority indicators.	Borderline Improved: Most indicators score 2, but one or more priority indicators score 1, trending toward Deficient.
**Deficient**	Most indicators score 1 or above, including all priority indicators.	Borderline Deficient: Most indicators/priority indicators score 1, but exactly one priority indicator is absent (= 0), placing the scenario on the immediate threshold of Critical.
**Critical**	Most indicators are absent (= 0), or two or more priority indicators are absent (= 0).	––––

“Most/majority” means more than half of the indicators evaluated for the criterion. Key indicators flag structurally important aspects: e.g., a single absent key indicator places the criterion in the deficient–critical transition, and two or more absent key indicators make it critical.

Each criterion is assigned to the highest scenario whose threshold it satisfies; when no threshold above Critical holds, the criterion is classified as Critical by default. A threshold combines two conditions. The first is a majority condition: more than half of the indicators evaluated for the criterion must reach the corresponding level. Indicators that could not be interpreted are excluded, so the majority is computed over assessed indicators only—and reaching exactly half is not a majority, which is itself sufficient to drop a criterion to the level below. The second is a gating condition on the priority indicators (Table 4)—the elements the SLR identified as recurrent and foundational across participatory processes (e.g., a formal legal framework, basic documentation). All priority indicators must themselves reach the level, so that a high rating cannot be obtained by accumulating points on peripheral indicators while core requirements remain unmet. Accordingly, Critical captures both the collapse of the majority (most indicators absent) and the failure of the structural core (two or more priority indicators scoring zero).

**Table 4:** Synthesis of the SysMAPP framework: mandatory indicators and performance requirements for Critical (0) and Advanced (3) levels. The codes in parentheses (e.g., I3) correspond to the indicator numbers in the complete evaluation matrix

Crit.	Total Ind.	Priority Ind. (n^o^)	What the “Advanced” (3) level requires?	What the “Critical” (0) level involves?
**Planning**	7	Legal framing (1); Member opinion on council’s operation (3); Compliance (4); Diverse participatory tools adoption (6)	The legal framework defines all items (objectives, rules, and election); more than 70% of members consider the functioning adequate; full compliance with bylaws; and participatory arenas are active and compliant.	Legal framework/bylaws define none of the items or do not exist; less than 30% consider functioning adequate; total non-compliance with rules; and no participatory arenas are adopted.
**Representativeness**	6	Legal provision of social quotas (9); Attendance in meetings (11); Composition of the council (12)	Legal quotas are established for IPLCs, women, and vulnerable groups; all segments maintain average attendance over 70%; and stakeholders predominate over public authorities with all critical groups represented.	No quotas or minimum seats are legally established; no segment maintains average attendance of 70% (or most show absent/minimal attendance); and public authorities predominate with critical groups unrepresented.
**Transparency**	6	Access to minutes (15); Public data (16); Feedback on proposals (17); Accountability (18)	Documentation is publicly available on the PA’s website; all management data is updated; feedback is systematic; and complete accountability is ensured annually.	Documentation does not exist or is not publicly accessible; no information is available on the website; no feedback mechanisms are adopted; and no accountability is provided.
**Influence**	7	Legal competences (21); Compliance (23); Council’s agenda alignment to the PA objectives (25); Proposal incorporation (26)	Legal mandate to provide opinions on all strategic items; full compliance with these obligations; 90% or more of meeting topics align with PA objectives; and proposals are mostly incorporated.	No legal mandate to formally provide opinions on strategic items; total non-compliance with framework obligations; less than 50% of topics align with PA objectives; and no incorporation of stakeholder proposals.
**Effectiveness**	8	Trust building (27); Stakeholder-driven revision of regulations (29); Management plan and zoning development (30); Cooperation (31); Formal agreements (32)	More than 70% of members perceive increased trust; more than 70% of technical requests are addressed; zoning incorporates all socioecological items; joint actions are recurring; and formal agreements are a core practice.	Less than 30% perceive contribution to trust; no participation outcomes in the legal or technical framework; zoning was not developed within the process; no joint actions implemented; and no agreements are adopted.

Crit.=Criteria; Tot. Ind.=Total Indicators.

Because the boundary between adjacent scenarios is rarely clear-cut, the matrix defines borderline transition zones rather than treating the levels as closed compartments. A criterion that meets the majority for a given level but whose priority indicators lag one step below it is read as borderline, at risk of dropping to the next lower scenario (e.g., a majority at Level 2 with a priority indicator at Level 1 is borderline Improved, trending toward Deficient). At the lowest functioning level, a criterion that holds the deficient majority but loses exactly one priority indicator (= 0) sits in the deficient–critical transition, on the immediate threshold of collapse. This graduated reading operationalizes a “realist horizon” that balances ideal participatory principles (see Introduction) with practical management constraints: it distinguishes a structurally sound arena with isolated weaknesses from one whose foundational mechanisms are failing, without forcing either into a rigid category. Table 4 presents the final SysMAPP structure, highlighting priority indicators, while the full matrix is provided as supplementary material.

### Demonstrating the Application of the SysMAPP in the Case Studies

The SysMAPP follows a four-phase protocol designed to be context-sensitive and results-oriented; in this study, the system was applied up to the third phase. Phase 1 involves data collection from management plans, legal documents, meeting minutes (107 from APAMLN and 31 from PNBC), and interviews with 40 council members (27 in Brazil and 13 in Spain), followed by selecting applicable indicators while excluding those unsuited to the specific context (e.g., ind. 19 and 34). In Phase 2, indicators are applied based on performance levels (0–3), and in Phase 3, criteria are classified into the quality levels (Table 3). Phase 4 translates these results into management interventions, ranging from operational adjustments (e.g., meeting frequency) to structural reforms (e.g., legal framework review).

Some indicators could not be assessed because the data required for their interpretation were unavailable—mainly due to missing interviews and incomplete participation records. As a result, the number of indicators effectively evaluated, and thus the maximum attainable score, varies across criteria and cases. Accordingly, percentages are calculated based on the indicators assessed in each instance rather than against a fixed maximum. The resulting categorization of each criterion should therefore be interpreted as approximate. The application of the SysMAPP revealed contrasting results between the case studies. Table 5 presents examples of indicator application and their respective justifications. The full application is provided in the supplementary data.

**Table 5:** Performance scenario of each criterion for both case studies (APAMLN and PNBC)

Crit.	Case	Scenario	Score (Σ/max; %)	Synthesis of the result (with example indicators)
**Planning**	APAMLN	Improved	13/18 (72.2%)	Strong legal-institutional framework with legally defined participation objectives and council structure (ind. 1 = 3). There is full compliance with regulatory provisions and meeting frequency (ind. 4 = 3), though points are lost due to limited support mechanisms (ind. 7 = 1).
	PNBC	Deficient	6/15 (40.0%)	The legal framework clearly defines participation objectives and council structure (ind. 1 = 3). However, operations collapse due to a systematic failure to comply with election cycles and meeting frequency in 8 of 19 analyzed years (ind. 4 = 1). The single zero falls on a non-priority indicator (ind. 7 = 0, support mechanisms), so the criterion remains Deficient.
**Representativeness**	APAMLN	Improved	12/15 (80.0%)	Parity-based composition includes diverse sectors from fisheries to oil (ind. 12 = 2). Reserved seats for Indigenous Peoples and Local Communities (IPLCs) are present, though no gender quotas exist (ind. 9 = 2). Average attendance exceeds 70% for most segments, though not for all (ind. 11 = 2).
	PNBC	Critical	2/12 (16.7%)	Structural collapse of representation. While gender balance is legally foreseen, no other inclusion quotas exist (ind. 9 = 1). Attendance is critically low, with three represented segments having never participated (ind. 11 = 0), causing a severe underrepresentation of resource-dependent groups like shellfish gatherers and artisanal salt workers (ind. 12 = 1).
**Transparency**	APAMLN	Improved	10/12 (83.3%)	Robust accountability practices anchored by systematic feedback protocols and public responses to over 600 proposals during the management plan development process (ind. 17 = 3). Minor gaps remain: meeting minutes are produced but only available upon request (ind. 15 = 2), and the official website lacks some legal PA/council information (ind. 16 = 2).
	PNBC	Deficient	4/12 (33.3%)	Transparency is restricted: meeting minutes are not made publicly available (ind. 15 = 1), and the official website provides only limited legal information (ind. 16 = 1). Furthermore, feedback and accountability protocols are non-systematic, provided only when explicitly requested by representatives (ind. 17 = 1).
**Influence**	APAMLN	Improved	14/18 (77.8%)	The council effectively fulfills its legal obligations and mandates (ind. 23 = 3), showing a very high proposal acceptance rate that represents 77.5% of the management plan (ind. 26 = 3). its main limitation is that its formal authority covers management instruments but does not extend to strategic issues like PA manager selection (ind. 21 = 2).
	PNBC	Deficient–Critical	7/18 (38.9%)	Broad formal authority (including budget and manager selection) exists on paper (ind. 21 = 3), but actual practice shows only partial compliance with legal obligations and consultative duties (ind. 23 = 1). It triggers a borderline warning on the threshold of Critical due to a systematic rejection of stakeholder autonomy: public authority proposals have never lost a vote (ind. 26 = 0).
**Effectiveness**	APAMLN	Improved	13/18 (72.2%)	Participation regularly translates into tangible management outcomes, showing recurring success in adapting fishing regulations and Environmental Impact Assessments (EIAs) to local reality (ind. 29 = 2). It shows a direct impact on zoning, expanding low-scale use zones to 67.28% of the territory (ind. 30 = 2).
	PNBC	Critical	2/12 (16.7%)	Tangible outcomes are minimal and fail to generate structural changes, leaving little measurable imprint on management. Two priority indicators are absent (ind. 29 = 0 and ind. 32 = 0), making the criterion Critical: tangible outcomes are effectively nonexistent—agreement is isolated (ind. 32 = 1), and social control rests on a single actor (ind. 33 = 1).

*Crit*. Criteria. Score (Σ) is the sum of the 0–3 scores for indicators interpreted per criterion. The attainable maximum (max) equals the number of assessed indicators multiplied by 3, accounting for data availability; percentages are calculated relative to this dynamic maximum.

### Interactions Among Criteria and Participation Improvement Milestones

**Fig. 4:**
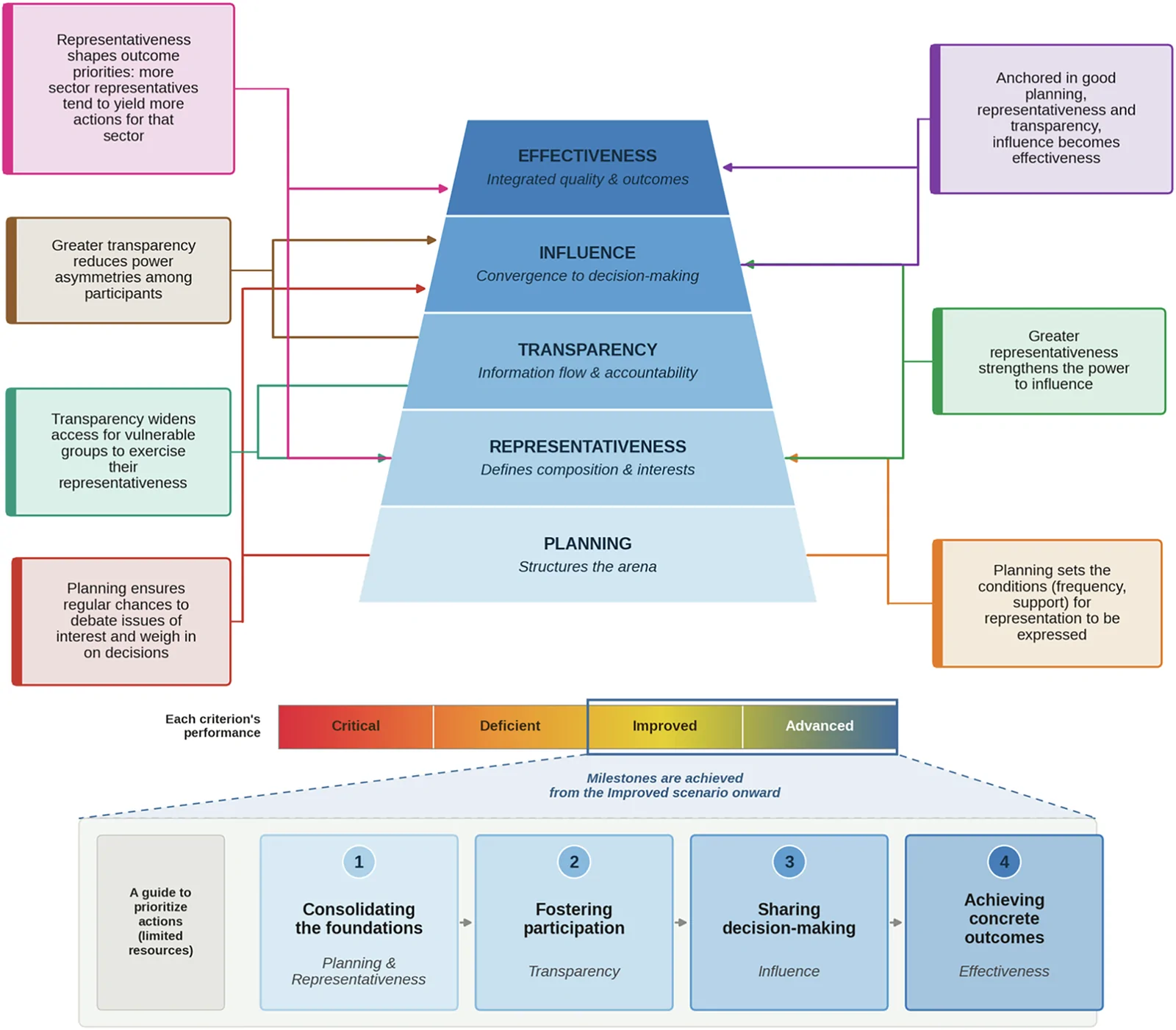
The SysMAPP framework: hierarchical ordering and interactions among the five criteria, and the link between criterion performance and participation-improvement milestones

The application of SysMAPP in the case studies revealed a hierarchical organization among its criteria, in which each performs a distinct function while directly conditioning the others. This organization is a central conceptual contribution of the system: by making the internal logic of participation explicit, it allows targeted adjustments to a given element whose effects then reverberate across the others (Fig. 4). At the base, Planning and Representativeness structure the participatory arrangement: Planning establishes the minimum conditions for the arena to operate, while Representativeness defines the council’s composition and thereby which interests are most likely to be raised, negotiated and prioritized downstream, in Influence and Effectiveness. Representativeness, in this sense, is not merely the presence of given segments but the configuration of a field in which certain actors can exert greater influence and secure benefits in the outcomes.

At the intermediate level, Transparency operates as the axis of information flow, accountability and feedback: it widens access for vulnerable groups (Representativeness), lets stakeholders monitor non-compliance in Planning through participation records (Gilliland and Laffoley, 2008; Quesada-Silva et al., 2019), and subjects state action to public scrutiny, curbing power concentration and clientelism (Arnstein, 1969; Borrini-Feyerabend and Jaeger, 2017). Influence then emerges as the moment of consummation of participation—the point at which the three preceding criteria converge into actual decision-making over the PA; when that moment is well grounded by the others, influence tends to raise Effectiveness. Crucially, real influence cannot be read off a council’s formal status: the APAMLN managing council and the PNBC council are both consultative bodies, yet they occupy clearly distinct positions on Arnstein’s (1969) ladder—a contrast that only the articulation of SysMAPP’s criteria makes visible. By the same logic, the mere existence of seats reserved for vulnerable groups does not make an arena inclusive; their effective presence, the support mechanisms available, and whether outcomes favor them must also be verified.

Effectiveness sits at the apex, expressing the integrated quality of the whole: outcomes consistent with the council’s social composition, built through planned and transparent processes, where influence moves beyond formal procedure into concrete changes aligned with management objectives and group demands. The clearest expression of this architecture is the chain Representativeness → Influence → Effectiveness: the cases show these criteria to be directly correlated, and the evidence suggests the relationship is also causal. In the APAMLN, a greater presence of IPLC (Representativeness) increased the incidence of artisanal-fisheries agendas (Influence) and led to the prioritization of this activity in zoning (Effectiveness), producing outcomes such as the management of spatial conflicts between artisanal and large-scale fisheries and accountability pressures over the impacts of oil, gas and port activities—impacts increasingly recognized in the port-sustainability and coastal-governance literature as falling within the responsibilities of port authorities (González-Laxe et al., 2025). The PNBC illustrates the chain in reverse: the absence of salt workers and shellfish harvesters from the council (Representativeness) leaves their concerns unadvocated (Influence), which in turn limits results on abandoned salt pans and illegal harvesting (Effectiveness); low Planning quality and weak Transparency further empty the process, discouraging engagement and feeding back into low Representativeness (Flannery et al., 2018; Cioffi et al., 2024). Effectiveness thus depends not only on Influence but, ultimately, on the foundational criteria that condition it.

Based on these findings, it is possible to propose adaptation milestones grounded in the relationships among the analytical criteria: (1) consolidating the foundations of participation; (2) fostering participation through a bidirectional flow of information; (3) sharing decision-making on issues of common interest; and (4) achieving concrete outcomes. These milestones correspond to the two highest levels of each criterion (Fig. 4) and serve as a guide to prioritize actions, especially in contexts of limited human, time, and financial resources, allowing adaptive measures to be strategically oriented toward their attainment. The milestones also acknowledge that if foundational criteria are not sufficiently met (Planning and Representativeness), achieving the upper stages is unlikely. This pattern is evident in the case studies: APAMLN reached all milestones, suggesting that adjustments in the participatory process can improve performance toward the advanced scenario, while PNBC did not surpass any of the defined milestones, highlighting the need to restructure its participatory process.

### Strengths and Limitations of the SysMAPP in Relation to Other Existing Tools

Beyond evaluating participation, the distinctive contribution of SysMAPP is that it operates as a tool for institutional diagnosis, accountability and adaptive governance. Because its criteria are hierarchically related, the System does not merely score a participatory arena: it diagnoses where the process underperforms and why, locating the specific criterion—and the chain it sets in motion—that constrains influence and outcomes. By making procedures, information flows, and decision-making power explicit and traceable, it provides an evidence base for holding management bodies accountable. Above all, it supports adaptive governance by occupying the evaluation stage of the management cycle: applied regularly as a monitoring and assessment instrument, its results feed back into management, allowing managers to track progress toward participatory objectives, identify failures and opportunities, and adjust strategies in successive cycles (Barragán Muñoz, 2014; Charnley and Engelbert, 2005; SINAC, 2016). The adaptation milestones operationalize this feedback loop, converting the diagnosis into prioritized, context-sensitive interventions even under resource constraints.

SysMAPP aligns with tools identified in the SLR by understanding participation as a means to achieve management outcomes, rather than as an end in itself. This is reflected in its structure: Representativeness and Influence are treated as conditions for decision-making, while Effectiveness focuses on concrete management responses. This differs from tools that frame aspects such as bottom-up approaches as outcomes (Etxano 2015; Davies et al., 2018; Paes, 2018; Ecuador Environmental Ministry, 2015). The System also advances beyond generic approaches, such as the “meaningful decision space” indicator (Rasheed and Abdulla 2020), by adopting detailed indicators and metrics to reduce subjectivity. Still, subjectivity cannot be fully eliminated, since evaluation depends on context and on different interpretations of adequate participation (Rowe and Frewer, 2000). For this reason, some metrics retain flexible terms such as “recurrent” or “scarce”.

A second strength is the integration of formal and practical dimensions of participation, supporting the finding that legal obligations alone do not ensure effective participation (Barragán Muñoz and Ruiz 2015). PNBC illustrates this, as it showed lower practical influence despite broader legal competences than APAMLN. This comprehensiveness enables precise contextual analysis. The System avoids, for instance, fixed meeting‑frequency thresholds, as both excessive meetings with irrelevant agendas and insufficient meetings to address stakeholders’ concerns can undermine engagement (Barragán Muñoz, 2014; Bockstael et al., 2016; Cioffi et al., 2024). For instance, although PROFONANPE (2007) recommends two annual meetings, evidence from PNBC shows this frequency is insufficient. Thus, the SysMAPP jointly evaluates the formalization, implementation, and perceived adequacy of meeting periodicity relative to PA demands. Also, compared to the most comprehensive tools identified in the SLR—Carrillo et al. (2023) and PROFONANPE (2007)—SysMAPP adopts a more integrative view of participation by incorporating key elements from both, such as co‑responsibility (operationalized under Influence) and managerial capacity (addressed within Planning), while Carrillo et al. (2023) omit Influence and Effectiveness, and PROFONANPE (2007) excludes Planning and Influence.

Finally, SysMAPP emphasizes participation as a means of social expression, aiming to counter lobbying pressures and state tutelage (Jacobi, 1996). Its Representativeness criterion prioritizes civil society and vulnerable groups, differing from parity-based models (WWF-Brasil, 2013) and recognizing the risk of PA instrumentalization by dominant interests (Andaloro and Tunesi, 2000; Labanino and Dobbins, 2023). By doing so, the SysMAPP helps prevent situations like those in APAMLN and PNBC, where mariculture lobbying threatened artisanal fishing in the zoning process and public authorities controlled participatory outcomes, respectively. Nevertheless, the System is mainly suited to formal and continuous participatory bodies, being less sensitive to sporadic mechanisms such as public hearings. It also does not replace stakeholder mapping or relational network analysis, requiring complementary tools such as power–interest matrices (Aguilar et al., 2012; Rubio et al., 2017). This design choice ensures continuous applicability by avoiding complex datasets and analysis, thereby excluding elements like “democratic recognition” (Davies et al., 2018; Paes, 2018) or “qualification levels” (Ospina et al., 2020). Consequently, the System is unable to verify claims such as those from PNBC authorities that stakeholder proposals were dismissed on technical grounds.

### SysMAPP as a Tool for Managers and Stakeholders: Usability and Replicability at Different Scales

SysMAPP indicators bridge technical rigor with practical usability, aligning with the quality attributes proposed by Bahia (2021) and supporting H2. By utilizing official documents (reliability), the system effectively assesses abstract criteria like Influence (representation), enabling the identification of citizen power levels (usefulness). For example, from the Arnstein (1969) perspective, Influence allows identifying a “partnership” level in APAMLN, while PNBC remains at a “symbolic” stage. Beyond these, the System ensures cost-effectiveness by using existing PA data and maintains stability by using institutional boundaries as analytical reference. Regarding simplicity, most indicators are straightforward, though those requiring averages or percentages demand some technical familiarity; however, technical barriers can be overcome through collaboration where skilled groups assist others in navigating bureaucratic and analytical requirements (Villi 2018).

The SysMAPP application also translates stakeholder perceptions into systematic assessments, contributing to the “materialization of social control” (Villi, 2018). In PNBC, the analysis of meeting minutes confirmed stakeholder perceptions of limited influence and resistance from public authorities. For managers, the System goes beyond operational checks, such as meeting format or document disclosure, by capturing engagement dynamics. By linking Representativeness and Influence indicators, participation in APAMLN was found to intensify during high‑stakes issues such as zoning disputes and oil and gas exploitation licensing, and to decline in routine meetings, reinforcing that engagement depends on issue relevance and perceived gains or losses (Buono et al., 2012; Flannery et al., 2018).

The System can also guide stakeholder prioritization where council seats are limited. In PNBC, it was identified that shellfish gatherers, salt workers and fishers remain unrepresented despite their vulnerability to management decisions and relevance for biodiversity conservation (de Andrés et al., 2018; Valle, 2023; Andalusian Government, 2024). It also revealed the absence of real estate and port infrastructure representatives, despite their critical role in salt‑marsh loss over the last six decades (De Andrés et al., 2018). De Andrés et al. (2018) show that the port and naval expansion eroded the bay’s erosion-control and disturbance-regulation services, corroborating a salt producer’s account that coastal-water erosion is the main pressure on the salt pans—demanding continuous and often financially unviable maintenance—and that the deteriorating quality of the water entering the marshes directly affects the salt produced. This gap is increasingly relevant: port authorities are shifting from a purely logistical and economic role to an ecosystem-based model with explicit responsibilities in environmental management, territorial governance, and the green transition (González-Laxe et al., 2025). Under the “green port” agenda, they are reframed as policymakers who act through corporate social responsibility, stakeholder collaboration, and participatory processes, placing community involvement at the core of port management (González-Laxe et al., 2025). In contrast, low‑relevance actors continue to hold council seats, revealing misalignment between representation and territorial impacts (e.g., consumer association).

Beyond PA councils, SysMAPP can be applied to other scopes and scales, provided that certain conditions are met: being institutionalized, continuous, and well-documented. Thus, beyond evaluation, it supports the documentation of participation by highlighting key aspects to be systemized. This effort shows how process quality translates into outcomes, as in APAMLN, where participation expanded small‑scale zones, ensuring regulatory coherence with the EEZ, which enhances stakeholder understanding, compliance, and enforcement (Cicin‑Sain and Belfiore 2005; Lewis et al., 2017).

## Conclusion

The SysMAPP offers a structured framework for assessing the quality of public participation in protected areas (PAs) through five hierarchically organized criteria: Planning and Representativeness define the operational and social foundations of participation; Transparency and Influence enable information flow, accountability and decision-making power; and Effectiveness captures concrete management outcomes. By translating these criteria into objective indicators, metrics and four interpretative levels—Critical, Deficient, Improved and Advanced—the System addresses methodological gaps identified in the literature, particularly the tendency of existing tools to focus either on procedural stages or on isolated outcomes, strengthening standardization, comparability and replicability across evaluators and institutional contexts.

Returning to the questions that guided the study, SysMAPP answers both. With respect to RQ1—how to design an MA model that preserves analytical rigor while adapting to diverse contexts—applying a single instrument to two markedly different settings (Brazil and Spain) showed that standardized, mandatory indicators can coexist with context-sensitive variables, enabling cross-case comparison without erasing local specificities; this supports H1. With respect to RQ2—how the systematic MA of participation can support legitimacy and adaptive management—assessing procedures and outcomes jointly exposed the conditions under which participation does or does not translate into influence and results: the hierarchical relationships among criteria, and the adaptation milestones derived from them, indicate where interventions are most likely to improve participation, as shown by the contrasting trajectories of the APAMLN and the PNBC. This supports H2 and represents the System’s central contribution: not merely measuring participation but explaining how its procedural quality conditions its outcomes and where to act.

The application also revealed three main limitations. First, several indicators depend on detailed documentation (e.g., management plans, legal instruments and meeting minutes), so the System’s applicability is reduced in PAs with sparse or poorly maintained records or limited technical capacity. This limitation affected the present study, as some indicators could not be assessed due to data unavailability—particularly where interview coverage or documentary evidence was incomplete—thereby constraining the overall completeness of the case study evaluations. Second, SysMAPP is designed for formally constituted, continuous participatory bodies such as councils and committees, and is therefore of limited applicability to informal or one-off mechanisms (e.g., public hearings, opinion surveys or diffuse forms of visitor participation) that lack continuity of debate and deliberation. Third, the System was applied only up to its third phase: Phase 4, which translates the results into management interventions, has not yet been tested. Future research should apply SysMAPP to a broader range of cases and test Phase 4, while the development of an application guide could further reduce operational barriers for managers and stakeholders.

Beyond its analytical function, SysMAPP aligns with contemporary agendas of democratic management, transparency and social control, reflected in international agreements such as the 2030 Agenda, the Escazú Agreement and the European Green Deal, which emphasize access to information, accountability and collective decision-making. A persistent gap remains, however, between the legal provision of participation and its effective operationalization in PAs—and it is precisely here that the System’s main opportunity lies: there is a concrete demand for instruments able to monitor and assess the quality of public participation, creating a favorable environment for tools that bring new inputs to management. SysMAPP can be institutionalized by managing agencies to support adaptive management and accountability, complementing existing monitoring and assessment mechanisms, while its flexibility broadens its potential use by a wide range of social actors as an instrument of social control to guarantee the right to participate and be heard in environmental matters.

## Supplementary information

Online Resource 1

Online Resource 1

Online Resource 1

## Data Availability

Detailed data supporting this study are available in the supplementary materials and the São Paulo State University institutional repository at https://hdl.handle.net/11449/319415. Interview data are not publicly available due to ethical and confidentiality restrictions, but anonymized materials may be made available from the corresponding author upon reasonable request.

## Declarations

### Use of generative AI

Gemini and Microsoft Copilot were used to enhance the vocabulary and readability of this work. The authors reviewed and edited the content as needed, taking full responsibility for the content of the publication.
