# Supplementary material for: A Model for Evaluating the Procedural and Effectiveness Dimensions of Public Participation in Protected Areas: The SysMAPP
Source: Environ Manage. 2026 Aug 29;76(9):294. doi: 10.1007/s00267-026-02603-0 (PMC13525943; doi:10.1007/s00267-026-02603-0)
Supplement: Supplementary file 1 [file 267_2026_2603_MOESM1_ESM.pdf]

Supplementary Data

Online Resource 1. Full SysMAPP matrix and scoring scheme

The System for Monitoring and Assessment of Public Participation (SysMAPP) is structured around five core criteria: Planning, Representativeness, Transparency, Influence, and Effectiveness. Each criterion is operationalized through indicators, and each indicator is assessed on a scale from 0 to 3, based on evidence-based scenarios of public participation in protected areas.

**Criterion: Planning** *(Priority indicators: 1 – 3 – 4 – 6)*				
**Indicator**	**0**	**1**	**2**	**3**
1 Legal definition of: (i) objectives of public participation for the PA; (ii) election of members and chair (when applicable); (iii) operating rules—frequency, records (minutes, reports), meeting format (in-person/online/hybrid), meeting disclosure	Legal framework/bylaws define none of the items or do not exist	Legal framework/bylaws define only one of the items	Legal framework/bylaws define participation objectives and election/selection procedures	Legal framework/bylaws define all items
2 Designing of tools (e.g., indicators) to periodically monitor the participatory body/process (or other participatory body) regarding: (i) composition; (ii) functioning; (iii) transparency; (iv) decision-making; (v) results	No tools for monitoring are provided	Tools address three or fewer items	Tools address four items	Tools address all items
3 Opinion of participatory body/process members regarding the meeting frequency, format, location and schedule	Less than 30% consider functioning adequate	Less than 50% consider functioning adequate	Between 50% and 70% consider functioning adequate	More than 70% consider functioning adequate
4 Compliance of participatory body/process functioning with the legal framework or bylaws (items ii and iii of Indicator 1)	Total non-compliance	Compliance with only one item	—	Full compliance
5 Presence of a specialized facilitator or formal training of the PA management team for mediation	No specialized facilitation and no formal training	—	No formal training, but self-initiated mediation training occurred	Specialized facilitation exists or formal institutional training was provided
6 Adoption of committees, commissions, working groups, or other participatory arenas to address specific topics within the participatory body/process	No participatory arenas adopted	Participatory arenas exist but most are inactive or non-compliant	Participatory arenas exist, partially active, and compliant	Participatory arenas exist, are active, and compliant
7 Enabling participation through: (i) logistical support; (ii) capacity-building and technical support (e.g., digital tools); (iii) gender-inclusive mechanisms	No enabling measures adopted	Enabling measures are rare	Enabling measures are provided when requested	All enabling measures are systematically adopted
**Criterion: Representativeness** *(Priority indicators: 9 – 11 – 12)*				
**Indicator**	**0**	**1**	**2**	**3**

8 Mapping, within the management plan or another management instrument, of PA stakeholders (institutions, associations, companies, and other organizations)	There is no mapping of PA stakeholders	—	The mapping exists but is outdated	The mapping exists and is up to date
9 Legal provision (e.g., PA creation decree or participatory body/process bylaws) for quotas/minim um number of seats to the representation of the following social groups: (i) Indigenous Peoples and Local Communities (IPLCs); (ii) women; (iii) other vulnerable social groups	No quotas/minimum number of seats are legally established	Quotas/minimum seats only for women	Quotas/minimum seats only for vulnerable social groups (no gender criterion)	Quotas/minimum seats for IPLCs, women and other vulnerable social groups
10 Opinion of participatory body/process members regarding representativeness	Less than 30% consider the participatory body/process representative	Less than 50% consider it representative	Between 50% and 70% consider it representative	More than 70% consider it representative
11 Average attendance[1] of representatives of each segment at meetings over one year or over the participatory body/process's mandate	No segment maintains average attendance ≥70%, or most segments show absent/minimal attendance (<30%)	Only a minority of segments maintain average attendance ≥70%, or some segments show absent/minimal attendance (<30%)	Most segments maintain average attendance ≥70%	All segments maintain average attendance ≥70%
12 Composition of the participatory body/process considering: (a) proportion between public authorities and stakeholders; (b) among stakeholders, presence of groups that: (i) hold management-relevant knowledge; (ii) are most affected by management actions; (iii) have historical or cultural ties to the territory; (iv) depend economically or socially on resource use; (v) have interests aligned with conservation and development goals; (vi) have high impact potential on the PA	Predominance of public authorities over stakeholders, and critical groups are not represented	Parity between public authorities and stakeholders, but some critical groups are not represented	Parity between public authorities and stakeholders, and all or critical groups are represented	Predominance of stakeholders over public authorities, and all critical groups are represented
13 Diversity of institutions/organizations represented in the participatory body/process[2]	Low institutional diversity (<0.3)	Regular institutional diversity (0.3–0.69)	High institutional diversity (0.7–0.99)	Maximum institutional diversity (=1)
**Criterion: Transparency** *(Priority indicators: 15 – 16 – 17 –18)*				
**Indicator**	**0**	**1**	**2**	**3**
14 Opinion of participatory body/process members regarding transparency	Less than 30% consider the participatory process transparent	Less than 50% consider it transparent	Between 50% and 70% consider it transparent	More than 70% consider it transparent

[1]**Average attendance** is calculated as the ratio between actual attendance (A_actual) and possible attendance (A_possible). A_possible corresponds to the number of seats allocated to a segment multiplied by the total number of meetings analyzed, while A_actual refers to the total number of attendances recorded for that segment across all meetings. For example, a segment with five seats and five meetings has a possible attendance of 25; if actual attendance totals 13, average attendance equals 0.52 (52%).

[2]**Institutional diversity** is calculated by dividing the number of institutions/organizations represented by the total number of seats. Values closer to 1 indicate higher institutional diversity, while values closer to 0 indicate lower diversity.

15 Documentation of the participatory process (minutes/reports) and public access	No documentation exists or is not publicly accessible	Documentation exists but access is subject to specific requirements, limiting public access	Documentation exists and is available to general public, but upon request	Documentation exists and is publicly available on the PA's website
16 Availability on the PA's website of: (i) legislation and regulations; (ii) management plan and associated content (diagnostics, zoning); (iii) news and deliberations	No information is available	Only one of the items is available or they are not regularly updated	At least two items are available and they are regularly updated	All items are available and updated
17 Adoption of formal feedback mechanisms on proposals, demands, and participatory results	No formal feedback mechanisms are adopted	Formal feedback mechanisms exist but are implemented irregularly or only when requested	—	Formal feedback mechanisms are implemented systematically and kept up to date
18 Accountability of the protected area management authority (reporting of actions, results, and resource use)	No accountability is provided by the management authority	Incomplete or irregular accountability, with no periodicity	Complete accountability ensured at least once per participatory body/process cycle	Complete accountability at least annually
19 Linguistic accessibility measures, including translation of relevant documents into languages used by IPLCs and simplification of technical language	No linguistic accessibility measures are adopted	—	Accessibility measures are adopted, but upon request	Accessibility measures are systematically adopted whenever essential documents are debated

Criterion: Influence *(Priority indicators: 21 – 23 – 25 – 26)*

Indicator	0	1	2	3
20 Legal establishment and decision-making nature of the participatory body/process	No participatory body is legally established	Participatory body is legally established with a consultative nature	—	Participatory body is legally established with a deliberative nature
21 Legal requirement for the participatory body to participate in decision-making on: (i) participation guidelines; (ii) management plan; (iii) appointment of the PA manager and allocation of financial resources; (iv) initiatives with high potential impacts on the PA	No legal mandate to formally provide opinions on the listed items	Legal mandate to formally provide opinions on one or two of the items	Legal mandate to formally provide opinions at least on three items	Legal mandate to formally provide opinions on all items
22 Opinion of participatory body/process members regarding influence	Less than 30% consider that they have influence over decision-making	Less than 50% consider that they have influence over decision-making	Between 50% and 70% consider that they have influence over decision-making	More than 70% consider that they have influence over decision-making
23 Compliance of participatory body/process with the obligation to issue opinions on legally required items (see Indicator 21)	Total non-compliance with items established in the PA's legal framework	Partial compliance with items established in the PA's legal framework	—	Full compliance with items established in the PA's legal framework
24 Shared leadership within the participatory body/process between public authorities and stakeholders	Stakeholders hold no leadership roles	Stakeholders rarely hold roles or lack autonomy	Stakeholders occasionally hold roles with autonomy	Stakeholders have formally defined roles with autonomy
25 Alignment between topics discussed in participatory body/process meetings and PA objectives	Less than 50% of the topics are aligned with the PA's objectives	Between 50% and 70% of the topics are aligned with the PA's objectives	70% or more of the topics are aligned with the PA's objectives	90% or more of the topics are aligned with the PA's objectives

26 Incorporation of proposals presented by stakeholders into plans, programs, and official documents	No incorporation of proposals submitted by stakeholders	Limited incorporation of proposals, and non-incorporated proposals are not or are rarely justified	Partial incorporation of proposals, and non-incorporated proposals are justified	Full or majority incorporation of proposals, and non-incorporated proposals are justified
**Criterion: Effectiveness** *(Priority indicators: 27 – 29 – 30 – 31 – 32)*				
**Indicator**	**0**	**1**	**2**	**3**
27 Opinion of participatory body/process members on the contribution of the body/process to building trust and support for the PA	Less than 30% consider that it has contributed to increasing stakeholders' trust in and support for the PA	Less than 50% consider that it has contributed to increasing stakeholders' trust in and support for the PA	Between 50% and 70% consider that it has contributed to increasing stakeholders' trust in and support for the PA	More than 70% consider that it has contributed to increasing stakeholders' trust in and support for the PA
28 Opinion of participatory body/process members on the contribution of the body/process to achieving PA objectives	Less than 30% consider that it has contributed to achieving PA objectives	Less than 50% consider that it has contributed to achieving PA objectives	Between 50% and 70% consider that it has contributed to achieving PA objectives	More than 70% consider that it has contributed to achieving PA objectives
29 Stakeholder-driven development, revision, or rejection of regulatory or technical instrument (policies, normative, and environmental studies etc.) aligned with PA objectives (based on registered requests)	No participation outcomes in the legal and technical framework	Less than 50% of registered requests were addressed	Between 50% and 70% requests were addressed	More than 70% requests were addressed
30 Development of the management plan and zoning including concrete measures: (i) expansion or creation of low-impact or small-scale use zones; (ii) establishment or expansion of no-take or strict protection zones; (iii) measures promoting benefits or access for vulnerable or traditional groups; (iv) formal assignment of responsibilities or co-management arrangements	The management plan and zoning were not developed within the participatory body/process	The management plan and zoning were developed within the participatory body/process, yet none of the items were incorporated as a result	The management plan and zoning include the items, but prioritize one over the others; responsibilities (item iv) are defined	The management plan and zoning incorporate all items
31 Cooperation among stakeholders and with public authorities in monitoring, enforcement, conservation, and use regulation	No joint actions implemented among stakeholders and with public authorities	Sporadic joint actions implemented, involving limited stakeholders or restricted to a single management theme	Recurring joint actions are implemented, but primarily between public authorities and stakeholders	Recurring joint actions are implemented among stakeholders and with public authorities across multiple management themes
32 Adoption of formal agreements for conflict mitigation within the participatory body/process	No agreements are adopted	Agreements are rare and/or informal	Agreements are recurrent but many are informal	Formal agreements are a core conflict-mitigation practice
33 Stakeholder oversight of public authority actions to ensure compliance with PA objectives and management plan guidelines	No records of stakeholder oversight actions (questioning, requests or complaints)	Oversight actions are recorded but occur sporadically, or involve few members, or do not generate formal responses from public authorities	Oversight actions are regularly recorded and shared among members, but lack systematic follow-up	Oversight actions are regularly recorded, shared among members, and systematically followed up through formal procedures

34 Allocation of environmental compensation and mitigation resources based on PA territorial priorities	Allocation of environmental compensation or mitigation resources shows no relation to priorities discussed within the participatory body/process	Resource allocation rarely reflects priorities or needs identified through the participatory body/process	Resource allocation frequently reflects priorities or needs identified through the participatory body/process	All resources are allocated according to priorities defined through the participatory process, supporting PA objectives and territorial management needs
