# Supplementary material for: A Model for Evaluating the Procedural and Effectiveness Dimensions of Public Participation in Protected Areas: The SysMAPP
Source: Environ Manage. 2026 Aug 29;76(9):294. doi: 10.1007/s00267-026-02603-0 (PMC13525943; doi:10.1007/s00267-026-02603-0)
Supplement: Supplementary file 2 [file 267_2026_2603_MOESM2_ESM.pdf]

**A Model for Evaluating the Procedural and Effectiveness Dimensions of Public Participation in Protected Areas: The SysMAPP**

Journal: Environmental Management

**Giovana Cioffi** (corresponding author[a]); **Davis Gruber Sansolo; Javier Garcia Sanabria**

[a]*Research Group of Environmental Planning and Coastal Management, Bioscience Institute - São Paulo State University, Infante Dom Henrique Square, 11330-900, São Vicente, São Paulo, Brazil*

[a]*Research Group on Integrated Coastal Zone Management, University of Cádiz, Doctor Gomez Ulla Avenue, 11001, Cádiz, Spain*

[a]*E-mail address: giovana.cioffi@unesp.br*

## Online Resource 2. Case study application

| Criterion | No. Indicator | APAMLN | PNBC | Justification |
|---|---|---|---|---|
| Planning | 1 | 3 | 3 | Both cases scored 3 because their legal or regulatory frameworks define the main elements required for the functioning of the participatory body, including rules on composition, procedures and meeting organization. |
| | 2 | 2 | 1 | APAMLN scored higher because its management instruments include more mechanisms related to monitoring and evaluation of the participatory body. PNBC scored lower because these tools are more limited and do not systematically cover process and outcome dimensions. |
| | 3 | — | — | This indicator could not be interpreted because this study was unable to interview all members of the APAMLN management council and the PNBC governing board. |
| | 4 | 3 | 1 | APAMLN scored 3 because the functioning of the management council generally complies with the rules established in its legal and regulatory framework. PNBC scored lower due to evidence of non-compliance with expected meetings frequency. |
| | 5 | 2 | — | This indicator could not be interpreted in the PNBC context because, although the governing board has a president, there are no data on whether mediation training or facilitation capacity was provided. |
| | 6 | 2 | 1 | APAMLN scored higher because it uses participatory arenas such as working groups or thematic spaces to address specific issues. PNBC scored lower because such mechanisms are not consistently used, with records showing only six references to working group meetings across 18 years of analyzed minute. |
| | 7 | 1 | 0 | APAMLN scored 1 because some support mechanisms exist, but they are limited and do not fully address logistical support, technical capacity-building or gender-sensitive inclusion. PNBC scored 0 because no evidence was identified of systematic measures to enable participation through these mechanisms. |
| Representativeness | 8 | 3 | 0 | In the APAMLN case, the publication date of the management plan, 2022, was considered, assuming that stakeholder mapping had been updated up to that year. |
| | 9 | 2 | 1 | APAMLN scored higher because its legal and institutional framework provides broader recognition of specific social groups, particularly traditional communities. PNBC received |

| Criterion | No. Indicator | APAMLN | PNBC | Justification |
|---|---|---|---|---|
| | | | | a lower score because provision for reserved seats just covers women, no including other vulnerable groups. |
| | 10 | — | — | This indicator could not be interpreted because this study was unable to interview all members of the APAMLN management council and the PNBC governing board. |
| | 11 | 2 | 0 | APAMLN scored 2 because, although the overall analysis shows recurrent lower attendance among some segments, there has been greater participation by these segments in recent years. PNBC scored 0 because most represented segments do not even reach the minimum required attendance. |
| | 12 | 2 | 1 | APAMLN scored higher because its council involves a broader range of socially relevant actors; however, this inclusion remains structured under a parity-based composition between public authorities and stakeholders. In contrast, PNBC scored lower because key resource-dependent groups—such as fishers, salt workers, and shellfish gatherers—are absent, while sectors with high ecosystem impacts (ports and real state) are underrepresented. |
| | 13 | 3 | — | This indicator could not be interpreted in the PNBC context because data on the exact composition of the governing board were not obtained; only the general composition established by legislation was available. |
| Transparency | 14 | — | — | This indicator could not be interpreted because this study was unable to interview all members of the APAMLN management council and the PNBC governing board. |
| | 15 | 2 | 1 | APAMLN scored higher because meeting minutes and participatory process records are properly documented and made publicly available upon simple request. PNBC scored lower because access to documentation is subject to a bureaucratic procedure requiring a formal request, justification, and the provision of additional information. |
| | 16 | 2 | 1 | APAMLN scored higher because the management plan and relevant deliberations is more accessible through institutional website. PNBC scored lower because only the management plan is available. |
| | 17 | 3 | 1 | To interpret this indicator, it was considered that APAMLN has formalized feedback procedures, such as the reporting protocol at the beginning of each meeting and the feedback matrix for proposals made during the management plan elaboration process. In PNBC, the score was based on statements from some interviewees indicating that feedback depends on representatives requesting it. |
| | 18 | 3 | 1 | APAMLN scored 3 because the managing body provides annual reports on actions, results and management developments within the participatory process. PNBC scored lower because accountability practices is not systematic. |
| | 19 | — | — | This indicator could not be interpreted due to the absence of systematized data allowing evaluation according to the metrics. |
| Influence | 20 | 1 | 1 | Both cases scored 1 because, although the participatory bodies are legally established, their decision-making nature remains as consultative. |
| | 21 | 2 | 3 | PNBC scored higher because its legal framework formally requires the governing board to issue opinions on a broader set of management instruments and decisions. APAMLN scored 2 because formal consultation obligations exist, but they do not cover all evaluated items such as manager election or budget. |
| | 22 | — | — | This indicator could not be interpreted because this study was unable to interview all members of the APAMLN management council and the PNBC governing board. |
| | 23 | 3 | 1 | APAMLN scored higher because the council fulfills its formal consultation and manifestation obligations. PNBC scored lower because legal obligations to obtain formal opinions in topics such as manager election was not respected. |
| | 24 | 3 | 1 | The score of 1 in the PNBC case is justified by the fact that, despite the existence of the position of president of the governing board, interviewees reported a lack of autonomy due to limitations imposed by public authorities. |
| | 25 | 2 | 1 | APAMLN scored higher because more than 70% of the topics discussed in council meetings are aligned with the objectives of the protected area. PNBC scored lower |

| Criterion | No. Indicator | APAMLN | PNBC | Justification |
|---|---|---|---|---|
| | | | | because meeting agendas show that over 35% of the topics are related to operational issues and protocols. |
| | 26 | 3 | 0 | The PNBC score was based exclusively on information contained in the minutes of the governing board meetings, since it was not possible to analyze the elaboration and revision process of the PORN and PRUG due to data unavailability. |
| Effectiveness | 27 | — | — | This indicator could not be interpreted because this study was unable to interview all members of the APAMLN management council and the PNBC governing board. |
| | 28 | — | — | This indicator could not be interpreted because this study was unable to interview all members of the APAMLN management council and the PNBC governing board. |
| | 29 | 2 | 0 | Although this indicator was not systematically analyzed, the reading of all APAMLN management council minutes suggests that this practice is common. However, there are statements indicating pending issues and matters that have not advanced, such as fishing huts and biodiversity protection. |
| | 30 | 2 | — | It was not possible to analyze the elaboration and revision process of the PORN and PRUG due to data unavailability. In APAMLN, the score was mainly due to a significant increase in zones for low scale uses, although zones dedicated to biodiversity protection decreased. |
| | 31 | 2 | 0 | No cooperation initiative was identified between public authorities and stakeholders. |
| | 32 | 2 | 1 | In the PNBC case, only one agreement was identified between public authorities and aquaculture representatives to address the issue of cormorants. In APAMLN, only one formalized agreement was identified, the Shared Management Agreement for Ilha das Couves, although interviews mentioned other agreements made before the approval of the management agreement in the management plan, suggesting that they were not formalized. |
| | 33 | 2 | 1 | The score assigned to APAMLN results from the fact that social control is recurrent, both in discourse and in actions, such as the formal request to remove concessions from the agenda. However, it is marked by the absence of formal actions by social actors in critical moments, such as the arbitrary interruption of the management council in 2017 by the state government. In PNBC, social control is exercised predominantly by a single representative, Ecologistas en Acción. |
| | 34 | 3 | — | APAMLN scored 3 because evidence indicates that environmental compensation and conditionality resources were strategically allocated in accordance with the territorial context and management demands of the protected area. This indicator was not assessed for PNBC, as it was not applicable to the protected area’s reality. |
