# Supplementary material for: A Model for Evaluating the Procedural and Effectiveness Dimensions of Public Participation in Protected Areas: The SysMAPP
Source: Environ Manage. 2026 Aug 29;76(9):294. doi: 10.1007/s00267-026-02603-0 (PMC13525943; doi:10.1007/s00267-026-02603-0)
Supplement: Supplementary file 3 [file 267_2026_2603_MOESM3_ESM.pdf]

A Model for Evaluating the Procedural and Effectiveness Dimensions of Public Participation in Protected Areas: The SysMAPP

Journal: Environmental Management

Giovana Cioffi (corresponding author[a]); **Davis Gruber Sansolo; Javier Garcia Sanabria**

[a]*Research Group of Environmental Planning and Coastal Management, Bioscience Institute - São Paulo State University, Infante Dom Henrique Square, 11330-900, São Vicente, São Paulo, Brazil*

[a]*Research Group on Integrated Coastal Zone Management, University of Cádiz, Doctor Gomez Ulla Avenue, 11001, Cádiz, Spain*

[a]*E-mail address: giovana.cioffi@unesp.br*

Online Resource 3. Analytical elements identified in the Systematic Literature Review (SLR)

Planning

Analytical element	Sources
Early public involvement	Rasheed and Abdulla, 2020; Davies et al., 2018
Diversity of topics on the agenda; meeting agendas	Dilascio et al., 2023; Souza et al., 2022
Promotion of skills and capacity development among council members to improve management	Sáenz-Chávez et al., 2020; Carrillo et al., 2023
Measures to ensure the participation of underrepresented groups	Buono et al., 2012; Paes, 2018; Souza, 2006
Well-defined and clear objectives of the participatory process for participants; existence of laws and regulations	Kovács et al., 2017; Macêdo, 2008
The committee acts according to its mission and complies with its work plan, developed based on PA priorities	Paes, 2018; PROFONANPE, 2007; Carrillo et al., 2023; Sáenz-Chávez et al., 2020
Independent facilitation team; trained team for participation	Kovács et al., 2017; Davies et al., 2018; Buono et al., 2012; Borrini-Feyerabend, 1997
Flexibility of the management council to rearrange internal processes and procedures in response to changing internal and external conditions	Paes, 2018
Clear parameters regarding the power of participation, degree of investment, definition of responsibilities and election of members; council bylaws or annual plan; how these are defined	Borrini-Feyerabend, 1997; Rasheed and Abdulla, 2020; Macêdo, 2008; Paes, 2018; Carrillo et al., 2023;

Analytical element	Sources
	WWF-Brasil, 2013; Stolton et al., 2023; Sáenz-Chávez et al., 2020
Frequency of meetings, type of meeting, ordinary or extraordinary, and meeting location	PROFONANPE, 2007; Dilascio et al., 2023; Kovács et al., 2017; Rasheed and Abdulla, 2020; KimDung et al., 2013; Sáenz-Chávez et al., 2020; Stolton et al., 2023; Carrillo, 2023
Sufficient time and resources allocated to the process, ensuring effectiveness and cost-benefit; time allocated for submitting comments, minimum one week	Kovács et al., 2017; Buono et al., 2012; Davies et al., 2018; Carrillo et al., 2023; WWF-Brasil, 2013; Sonet et al., 2020
Type and diversity of participatory tools; creation of sub-councils, committees or special commissions to address specific topics and resolve conflicts or problems	Kovács et al., 2017; Buono et al., 2012; Sonet et al., 2021; WWF-Brasil, 2013; Sáenz-Chávez et al., 2020
Legal guarantee of the right to participate and voice for all local actors in PA management; collegiate participation body	Macêdo, 2008; Maestro et al., 2022; Getzner et al., 2012; SINAC, 2016
Council bylaws include evaluation of council functioning; periodic council evaluation, report production and revision of bylaws; factors that hindered or helped the process	Carrillo et al., 2023; Sáenz-Chávez et al., 2020; WWF-Brasil, 2013
Council bylaws include a mechanism to ensure compliance with agreements; monitoring of agreements and commitments per meeting	Carrillo et al., 2023; Sáenz-Chávez et al., 2020
Free, prior and informed consent processes are followed	Stolton et al., 2023

Representativeness

Analytical element	Sources
Representatives of all important stakeholders participate in the participatory process; equity in the creation process of protected areas; legitimacy	Kovács et al., 2017; Buono et al., 2012; Davies et al., 2018; PROFONANPE, 2007; Sáenz-Chávez et al., 2020; WWF-Brasil, 2013; Macêdo, 2008
Council representativeness of different sectors is proportional to their degree of incidence in the PA	Carrillo et al., 2023
The management council is recognized as a democratic representative of territorial interests	Paes, 2018
Park management has a good understanding of the historical, cultural and social qualities of the national park	Kaltenborn et al., 2011
People working in the PA recognize and consider the interests of Indigenous Peoples and Local Communities, IP&LCs, in the protected area system	Stolton et al., 2023
Council members act with commitment and demonstrate acceptance of their responsibilities	Paes, 2018; Borrini-Feyerabend, 1997

Analytical element	Sources
Qualification of strategic actors	Ospina Moreno et al., 2020
Number of sector representatives on the council; number and diversity of represented institutions; direct actors, groups whose productive activities and housing depend on PA resources, and indirect actors, groups outside the PA that influence its conservation and management	Souza et al., 2022; Dilascio et al., 2023; Carrillo et al., 2023
Number of people present	Dilascio et al., 2023
Inclusion of leaders and/or vulnerable groups; number of community representatives; degree of involvement	Souza et al., 2022; Buono et al., 2012; Dilascio et al., 2023; KimDung et al., 2013
Council bylaws define quotas or participation percentages for each sector	Carrillo et al., 2023
Council bylaws establish quotas or a number of seats for the representation of women, youth, Indigenous peoples, native peoples and Afro-descendant populations	Carrillo et al., 2023
The advisory council has a gender-sensitive inclusion strategy or mechanism	Carrillo et al., 2023
Is the council composition parity-based?	WWF-Brasil, 2013
Indigenous Peoples, Local Communities and other local actors are represented in decision-making bodies at higher levels, beyond local advisory committees	Stolton et al., 2023
Members of the advisory council receive training on equity issues from a gender perspective	Carrillo et al., 2023

Transparency

Analytical element	Sources
The process is transparent, allowing stakeholders to follow and understand how decisions are made; decision-making is more transparent and fairer	Kovács et al., 2017; Rasheed and Abdulla, 2020; Stolton et al., 2023; Maestro et al., 2022
Complaints and comments are addressed effectively	Souza et al., 2022
Communication mechanisms between actors and managers; periodicity; level of communication	Borrini-Feyerabend, 1997; Souza et al., 2022; Maestro et al., 2022; García and Mendes, 2019; Buono et al., 2012; KimDung et al., 2013; WWF-Brasil, 2013; PROFONANPE, 2007; Souza, 2006; Sáenz-Chávez et al., 2020
Council members know the meeting agenda in advance	Carrillo et al., 2023
Documentation of the participatory process; prior dissemination of meeting minutes	KimDung et al., 2013; Carrillo et al., 2023
Information on the PA is available to stakeholders and the general public, including diagnostics on conservation status, socioeconomic conditions, legislation and rules, management plans, zoning, PA performance and progress	Maestro et al., 2022; Rossi et al., 2024; Davies et al., 2018; Macêdo, 2008; PROFONANPE, 2007

Analytical element	Sources
Information on public hearings, roundtables and stakeholder committees aimed at public participation in decisions on projects, plans or regulations	Rossi et al., 2024; Sonet et al., 2021; Rasheed and Abdulla, 2020
Accountability; annual accountability	Paes, 2018; WWF-Brasil, 2013; PROFONANPE, 2007
Information on public participation results and final decision; feedback mechanisms	Rossi et al., 2024; Paes, 2018; Carrillo et al., 2023; KimDung et al., 2013
Information on mechanisms to challenge decisions on projects requiring public participation, and on illegal acts and omissions harmful to PA objectives	Borrini-Feyerabend, 1997; Rossi et al., 2024; Rasheed and Abdulla, 2020
Information on ongoing and/or past judicial proceedings involving the PA	Rossi et al., 2024

Influence

Analytical element	Sources
Bottom-up approach allowing the public to participate in management decisions; whether the local community living inside or near the park influences park decisions; extent to which tourism operators feel able to influence management issues; whether council dynamics influenced institutional action; identification of the most influential actors	Etxano et al., 2015; Davies et al., 2018; Paes, 2018; Ecuador, 2015; Buono et al., 2012; Kaltenborn et al., 2011; WWF-Brasil, 2013; Carrillo et al., 2023; Rastogi et al., 2010
Development of council referrals according to each sector	Souza et al., 2022
Respect and attention to different viewpoints; opportunity for dialogue; courtesy or absence of verbal or non-verbal devaluing behavior	Souza et al., 2022; Rasheed and Abdulla, 2020
Council members feel part of agreements, proposals and actions	Carrillo et al., 2023
The council plenary makes its own decisions, and its operation does not depend on the will of the PA management authority	Carrillo et al., 2023
Diversity of topics raised by social actors	Dilascio et al., 2023
Participation is guaranteed in such a way that stakeholders perceive the existence of real alternatives	Kovács et al., 2017
Shared leadership	Getzner et al., 2012
Search for consensus in mediating conflicting proposals or interests, aiming at the local common good	Macêdo, 2008
Inclusion of new agenda items in meetings by representatives other than PA management	Dilascio et al., 2023
Moments in which caiçara communities expressed their opinions	Dilascio et al., 2023
Number of deliberations in each meeting	Dilascio et al., 2023
Number of topics raised by social actors	Dilascio et al., 2023
Tourism operators’ perception of whether park managers serve the general public or favor specific interest groups	Kaltenborn et al., 2011

Analytical element	Sources
Represented sectors can decide on the composition and structure of the council	Carrillo et al., 2023
Number of proposals made versus incorporated	Turques de Andrade et al., 2007; Kovács et al., 2017
Participation in management rules, PA location, zoning, management plan and implementation	Zeng et al., 2022; Maestro et al., 2022; Macêdo, 2008; Sáenz-Chávez et al., 2020
Decision-making property of participation: deliberative or consultative	Souza, 2006

Effectiveness

Analytical element	Sources
Whether relationships among council members and partners helped or hindered progress in PA functioning	WWF-Brasil, 2013
Multidirectional learning	Davies et al., 2018; Paes, 2018
Differences and conflicts among actors with different interests and ideas were effectively resolved; level of conflict management	Ma et al., 2023; Sáenz-Chávez et al., 2020; Kovács et al., 2017
Community participation provides greater support for decision-making management and for the PA	Ma et al., 2023; Rastogi et al., 2010; Getzner et al., 2012; KimDung et al., 2013
Collaboration between PA management and actors; level of collaboration; collaboration for conservation actions	Kaltenborn et al., 2011; KimDung et al., 2013; Getzner et al., 2012; Rasheed and Abdulla, 2020; Ecuador, 2015; PROFONANPE, 2007; Sáenz-Chávez et al., 2020
Combination of multiple forms of knowledge	Davies et al., 2018; Kovács et al., 2017
Building trust in relation to the PA	Davies et al., 2018; Macêdo, 2008
Awareness about the PA	Dilascio et al., 2023; Rastogi et al., 2010
Promotion of the elaboration and implementation of consensual agreements; number of agreements defined in meetings; expansion of agreements over time, both geographically and in complexity	Sáenz-Chávez et al., 2020; Dilascio et al., 2023; Borrini-Feyerabend, 1997
Participation in environmental monitoring	Zeng et al., 2022
Participation in social monitoring, enforcement or oversight	Zeng et al., 2022
The council is considered useful by its members	Carrillo et al., 2023
New response policies, such as payment schemes, incentives or regulatory changes, emerged during the process	Kovács et al., 2017
Creation of compensation mechanisms and/or options for area use, promoting balance among PA objectives; practices that achieve a favorable cost-benefit balance for the local population	Macêdo, 2008
Council members consider the council a space to build and expand their relationship networks	WWF-Brasil, 2013
The participatory process was reviewed in terms of its effectiveness	Stolton et al., 2023
